# Evidence of Small Changes in Daytime Body Temperature in Active Black‐Capped Chickadees in Response to Supplemental Food Availability

**DOI:** 10.1002/ece3.73946

**Published:** 2026-07-05

**Authors:** Deborah M. Hawkshaw, Kimberley J. Mathot

**Affiliations:** ^1^ Department of Biological Sciences University of Alberta Edmonton Alberta Canada; ^2^ Canada Research Chair, Integrative Ecology, Department of Biological Sciences University of Alberta Edmonton Alberta Canada

**Keywords:** active‐phase, body temperature, diurnal thermoregulation, food availability, heterothermy, winter ecology

## Abstract

In winter, small birds may be able to reduce their energy expenditure by decreasing daytime body temperature (*T*_b_). While the use of reduced *T*_b_ during the active‐phase is not well understood, one factor predicted to shape *T*_b_ and heterothermy is food availability. We experimentally manipulated the presence of supplemental food at a bird feeder and monitored subcutaneous temperature (*T*_sub_) in 15 overwintering black‐capped chickadees ( *Poecile atricapillus* ) during feeder visits to investigate whether food availability shapes daytime *T*_sub_. Chickadees had lower *T*_sub_ when the ambient temperature was lower, and this effect was strongest when the feeder was empty. Diurnal patterns in *T*_sub_ also varied as a function of food treatment; on days when the feeder was full, chickadees exhibited a mid‐afternoon peak in *T*_sub_, while on days when the feeder was empty, chickadees exhibited a mid‐morning minimum in *T*_sub_. Treatment‐related differences in diurnal *T*_sub_ patterns and ambient temperature effects align with effects reported in earlier studies, though they are notably smaller in magnitude. We attribute the differences in effect sizes to the fact that our manipulations involved removal of supplemental food, rather than complete food deprivation, and that we measured *T*_sub_ in active individuals. Although the observed effect sizes for *T*_sub_ were small, this study is an important step toward understanding how access to a reliable food source during winter may shape energy management in small wintering birds. Future studies that obtain measures of *T*_b_ outside of when individuals are actively foraging are needed to elucidate how food availability shapes the overall use of *T*_b_ adjustments as an energy management strategy.

## Graphical abstract

We experimentally manipulated the presence of supplemental food at a bird feeder and monitored subcutaneous temperature (*T*_sub_) in 15 overwintering black‐capped chickadees ( *Poecile atricapillus* ) during feeder visits to investigate whether food availability shapes daytime *T*_sub_. We observed treatment‐related differences in diurnal *T*_sub_ patterns and ambient temperature effects that aligned with effects reported in earlier studies, though they were notably smaller in magnitude.

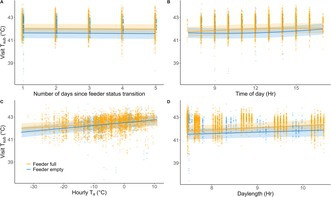

## 1 Introduction

In temperate regions, overwinter environmental conditions pose an energetic challenge to small non‐migratory bird species. The co‐occurrence of low ambient temperatures, reduced environmental food availability, and shorter daylengths (for diurnal species) means that increased thermoregulatory costs coincide with environmental constraints on energy acquisition. As such, for small birds in winter, the effective management of daily energy budgets is critical for survival. There are several strategies and seasonal adjustments that small birds use to survive these conditions (e.g., Chaplin 1974; Rogers and Rogers 1990; Petit et al. 2013; Broggi et al. 2019). For example, several species of small birds reduce their metabolic rate and body temperature (*T*_b_) below euthermic levels during the rest‐phase. This strategy is often termed “shallow torpor” in small passerines as reductions in *T*_b_ are generally shallow (< 10°C) compared to deeper bouts of torpor observed in other avian species (McKechnie et al. 2023). The extent to which shallow torpor is used has been shown to vary in response to energetic challenges including lower ambient temperature (Chaplin 1976; Nord et al. 2009) and reduced food availability (Nilsson et al. 2020). Some studies have also reported interactive effects between ambient temperature and food availability in terms of the extent to which is *T*_b_ reduced (e.g., Laurila and Hohtola 2005; Doucette et al. 2012) and torpor bout duration (e.g., Doucette et al. 2012). Although several species have been shown to exhibit reduced *T*_b_ during the active phase, reductions in daytime *T*_b_ are generally shallower compared to nocturnal reductions (Lewden et al. 2014), and have been less commonly studied (but see Butler and Woakes 2001; Laurila and Hohtola 2005; Laurila et al. 2005; Carere et al. 2010; Lewden et al. 2014; Hawkshaw et al. 2025). As such, our current understanding of the occurrence of reductions in daytime *T*_b_ are limited, including how daytime *T*_b_ may vary in response to the combined challenges of low ambient temperature and low food availability.

Our limited understanding of the use of low daytime *T*_b_ stems in part from challenges in measuring daytime *T*_b_. Measures of *T*_b_ during the day in active birds may reflect not only changes in *T*_b_ as a potential energy conservation strategy but can also capture changes in heat production due to activity (e.g., flight) and digestion. For example, in snow buntings ( *Plectrophenax nivalis* ) engaging in flight leads to an increase in *T*_b_ (O'Connor et al. 2024). Additionally, despite the potential energetic benefits of lowering *T*_b_ during the day, theoretical models predict this strategy should be avoided due to potential costs associated with reduced *T*_b_ [e.g., decreased vigilance (Amo et al. 2011); decreased manoeuverability when evading predators (Carr and Lima 2013); reduced foraging efficiency (Brodin et al. 2017)]. Indeed, these costs are thought to explain why reductions in daytime *T*_b_ are generally shallow (Lewden et al. 2014). However, evidence of low daytime *T*_b_ reducing foraging efficiency is lacking, and at least in black‐capped chickadees ( *Poecile atricapillus* ), increased foraging activity, as inferred from shorter intervals between successive feeder visits, are associated with lower daytime *T*_b_ (Hawkshaw et al. 2025).

Despite the challenges associated with measuring daytime *T*_b_, at least two empirical studies have documented daytime *T*_b_ below euthermic values in black‐capped chickadees, particularly at low ambient temperatures (Lewden et al. 2014; Hawkshaw et al. 2025). Although daytime *T*_b_ is not reduced to the same level as observed during the rest‐phase, the extent of reduction in daytime *T*_b_ is not related to an individual's thermogenic capacity (Lewden et al. 2014). This suggests the *T*_b_ reductions do not reflect an inability to generate sufficient heat to maintain *T*_b_ at low ambient temperatures, but rather, likely reflect an energy saving strategy similar to shallow nocturnal torpor or reduced regulation of *T*_b_ in response lower ambient temperature. Here, we evaluated whether experimental manipulations of food availability alter daytime *T*_b_ in free‐living black‐capped chickadees, and whether the effects of food availability on *T*_b_ are dependent on other factors known to shape daytime *T*_b_, including ambient temperature (Laurila and Hohtola 2005; Lewden et al. 2014; Winder et al. 2020), daylength (Laurila et al. 2005) and time of day (Lewden et al. 2014; Winder et al. 2020).

Most of our understanding of the role of food availability in shaping torpor and facultative hypothermia in birds has come from studies focused on rest‐phase *T*_b_ and/or metabolic rates in captive or semi‐captive studies (e.g., Reinertsen and Haftorn 1986; Hiebert 1990; Powers et al. 2003) (for reviews see Reinertsen 1996; McKechnie and Lovegrove 2002; Schleucher 2004; McKechnie et al. 2023), though observational (e.g., Doucette et al. 2012) and experimental (Nilsson et al. 2020) field studies do exist. Generally, such studies have found that when food resources are more limited (e.g., due to food restriction in captive settings, or low environmental food availability in field studies), this leads to an increase in the use of torpor (depth, duration). At least one captive study found that pigeons ( *Columba livia* ) exhibit a reduced daytime *T*_b_ in response to fasting, though only at the lowest ambient temperatures observed in the study (circa −25°C; Laurila and Hohtola 2005). We are aware of only one field study that has investigated peripheral thermoregulation (bill and eye temperatures) in response to food availability during the active phase in a small wintering bird. In that study, active great tits ( *Parus major* ) had lower bill temperatures when food was restricted (Winder et al. 2020), though this effect came about in part due to changes in the diurnal pattern of bill temperatures. Under *ad libitum* food availability, great tits exhibited a mid‐day peak in bill temperature, while under food restriction, great tits exhibited a mid‐day minimum in bill temperature (Winder et al. 2020). To date, no studies have experimentally investigated whether daytime *T*_b_ is shaped by food availability under field conditions. In order to understand the ecological contexts in which small birds in winter may exhibit lower *T*_b_, experimental investigations in field settings are needed (McKechnie et al. 2023).

Here, we experimentally manipulated the presence of supplemental food at bird feeders to evaluate whether food availability shapes daytime *T*_b_ in overwintering black‐capped chickadees. Black‐capped chickadees are an excellent system to address this question because chickadees are known to have lower daytime *T*_b_ under natural winter conditions (Lewden et al. 2014). Chickadees also readily use bird feeders, and access to such feeders enhances overwinter survival, suggesting that access to bird feeders provides a meaningful increase in energy intake compared to feeding exclusively on naturally available food (Brittingham and Temple 1988; Desrochers et al. 1988). In a single winter (Dec 2023—Feb 2024), we alternated between periods where supplemental food was available at bird feeders or not and recorded the subcutaneous body temperature (*T*_sub_) of marked individuals during feeder visits, which was possible because chickadees regularly visit feeders even when they are empty (Haave‐Audet et al. 2024). If low *T*_b_ during the day is used as a strategy to limit energy expenditure during energetic shortfalls, we predicted that during periods when supplemental food was not available at the feeder, individuals would have lower daytime *T*_sub_ compared to when supplemental food was available at the feeder. We also predicted that this effect would be greatest at lower ambient temperatures when the risk of energetic shortfall is highest. We also evaluated whether diurnal *T*_sub_ patterns differed as a function of supplemental food availability, following Winder et al. (2020). We predicted that the midday peak in body temperature would be higher under *ad libitum* supplemental food availability compared to when only natural food sources were available.

## 2 Material and Methods

### 2.1 Study System

We studied black‐capped chickadees at the University of Alberta Botanic Garden (UABG), Devon, Alberta, Canada (53° 24′ 27″ N, 113° 45′ 41″ W) from 25 November 2023 to 24 February 2024. Habitat within the garden consists of several manicured gardens (with a total area of 0.32 km^2^) and mixed deciduous forest (total area 0.65 km^2^). A marked population of black‐capped chickadees (hereafter, “chickadees”) has been maintained since Fall 2017 as a part of an ongoing study investigating chickadee overwintering ecology. Details on the design and capture protocols used at this study site can be found in Arteaga‐Torres et al. (2020). But briefly, at first capture each individual receives a unique passive integrated transponder (PIT) tag that can be detected at bird feeders equipped with radio frequency identification (RFID) antennas and readers. When a PIT‐tagged individual visits the feeder, their identity and the date and time of their visit are recorded to an SD card. In total, there are eight feeders distributed throughout the forested areas of the botanic garden with a minimum distance of 270 m between feeders. Minimum distances between feeders were based on previously reported overwinter flock territory sizes (Smith 1991), so that approximately one flock is monitored per feeder location. Individuals in the study population are sexed molecularly via a small blood sample (Griffiths et al. 1998) or via a discriminant function that was developed for the same population (Sridharan 2021), and are aged according to the appearance of their rectrices (Pyle 1987).

Between 25 November and 01 December 2023, we implanted individuals (*N* = 21) subcutaneously with temperature‐sensing PIT tags (precision 0.1°C, accuracy ±0.5°C; BioTherm13, Biomark, Boise, ID, USA). Prior to implanting tags, we confirmed the accuracy of each tag at temperatures ranging from 25°C to 46°C (see Tables S1 and S2). This temperature range for validations was chosen based on *T*_sub_ recordings previously found using this method in chickadees (Hawkshaw et al. 2025). Our study was a within‐subject design, whereby each variable of interest varied at the within‐individual level. Therefore, we did not apply a separate calibration function to each individual tag because the minimum detectable within‐subject effect size is limited by tag precision (i.e., 0.1°C). This means the minimum detectable effect size for all the within‐subject effects considered is 0.1°C across the full range of the factor of interest. For example, the minimum detectable effect of food supplementation treatment (categorical with 2 levels) was 0.1°C, while for ambient temperature, the minimum detectable effect was 0.1°C across the 44.9°C range in ambient temperature (i.e., between −34.0°C and 10.9°C, the minimum and maximum *T*_a_ values in our dataset, respectively).

PIT tags were implanted above the scapula following protocols described in Farr et al. (2021). In addition to these *N* = 21 birds, any birds which had been implanted the previous winter as part of another study (Hawkshaw et al. 2025), and which were detected in the current study year at the time of experiments (*N* = 7) were also included in this study. Of the 21 birds implanted in the current study year, one individual had been implanted in the previous year but subsequently lost its thermal tag so was re‐implanted with a new tag. We chose to implant the tags subcutaneously rather than intraperitoneally as previous work has shown that subcutaneous and intraperitoneal (core) measures of temperature do not differ significantly in small birds across a range of ambient temperatures (e.g., Great tits, *Parus major* , 15–20 g, ambient temperatures −15°C—25°C, Andreasson et al. 2023; and Zebra finches, *Taeniopygia guttata*, 15 g, ambient temperature 5°C—40°C, Oswald et al. 2018). Furthermore, in small (< 25 g) birds, injury rates for intraperitoneal implants are higher than for subcutaneous implants (e.g., 18.2% vs. 0% in Zebra finches, 15 g, Oswald et al. 2018). The minimum *T*_a_ recorded in this study (−34°C) was lower than the minimum *T*_a_ in the comparisons between subcutaneous and core temperatures carried out by Andreasson et al. (2023) in great tits, thus, we cannot rule out that subcutaneous temperatures may not mirror core temperatures across the full range of *T*_a_ in our study, and we therefore refer to our body temperature measures as *T*_sub_ (subcutaneous temperature) throughout.

The thermal PIT tags used to measure *T*_sub_ operate on a different frequency (134 kHz, Biomark Biotherm 13 PIT tag) compared to another type of PIT tag used in the same study population that does not register temperature (125 kHz, Eccel Technologies EM4102 PIT tag) and therefore required different RFID equipment (antenna and reader) to be detected. As such, we only implanted and monitored the *T*_sub_ of chickadees at one feeder location within the study site, while the remaining seven feeders were equipped with RFID antennas and readers to detect non‐thermal PIT tags. To detect the thermal tags at the “thermal” feeder, the feeder was equipped with a 7.62 cm circular antenna connected to a small‐scale monitoring system (Biomark, Boise, ID, USA). The antenna was positioned such that it could be used as a perch to retrieve black‐oil sunflower seeds from a small opening in the feeder. The antenna sensitivity was adjusted so that individuals were only detected when within close proximity (~7.62 cm from the antenna) and the reader was programmed to detect unique tags once every second.

### 2.2 Supplemental Food Availability Experiment

**FIGURE 1:**
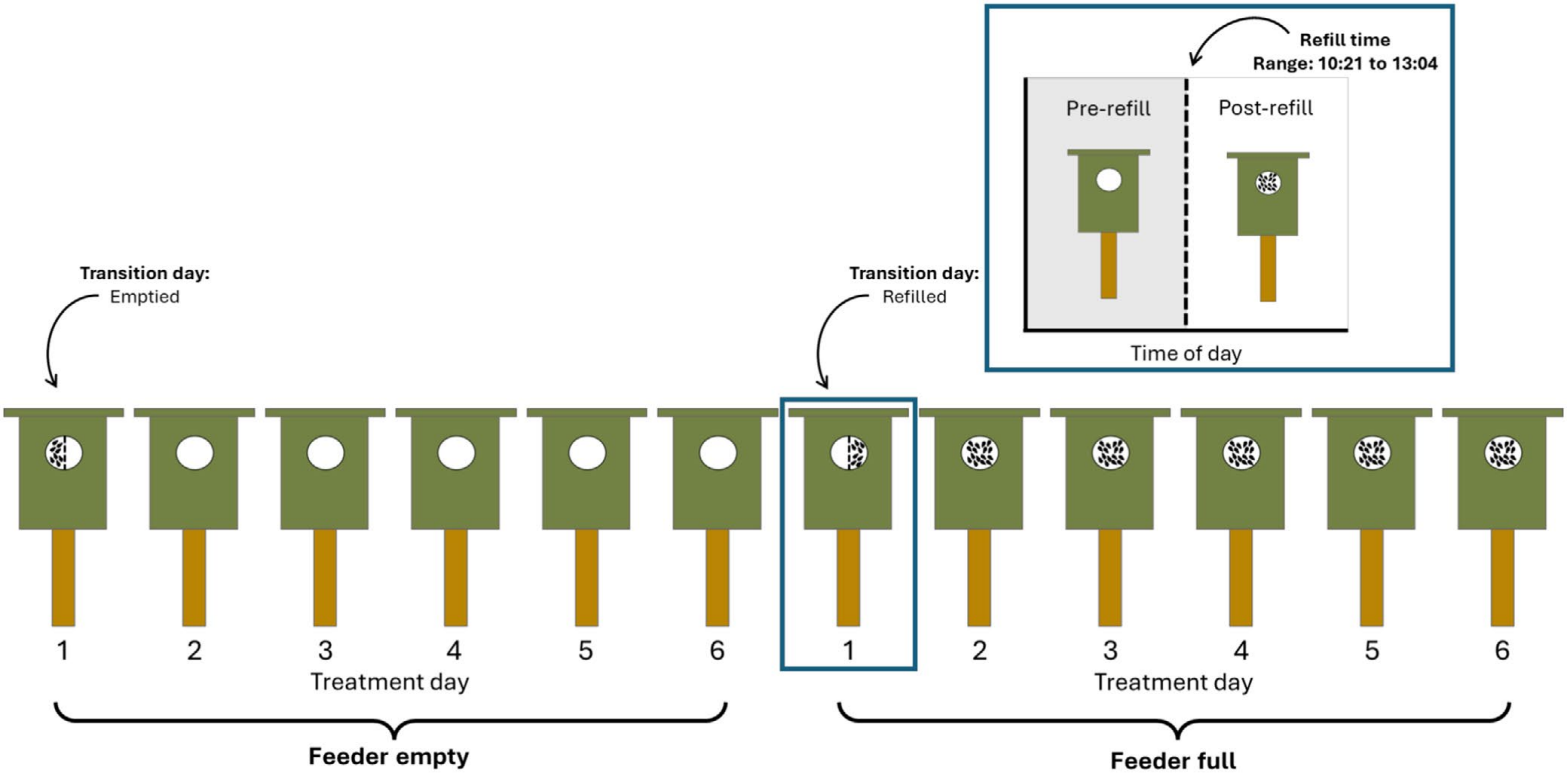
Schematic of replicate design for the present study evaluating effects of manipulating food availability at a supplemental bird feeder on active daytime *T*_sub_ in black‐capped chickadees. A replicate consisted of two treatments where the feeder was first empty for 6 days and then full for 6 days. The first day of a treatment was a transition day where the feeder was either emptied (for feeder empty treatment) or refilled (for feeder full treatment) part‐way through the day. The specific timing of when the feeder was emptied and refilled varied across replicates and are indicated in Table S3. The study consisted of six replicates in total. The blue inset indicates the timing of refills on transition days when the feeder was refilled, which across replicates ranged from 10:21 to 13:04.

To assess the effect of supplemental food availability on daytime *T*_sub_ we experimentally manipulated the presence/absence of supplemental food at each feeder in the garden. Experiments commenced 14 days after the last catching date to reduce potential carry‐over effects. During the period between catching and the start of experiments, the feeders were continuously filled with black‐oil sunflower seeds. Starting on 15 December 2023, we alternated between six‐day periods where chickadees only had access to naturally occurring food (feeder empty) and six‐day periods where chickadees had access to *ad libitum* supplemental food at the bird feeders (feeder full). The feeder was always filled or emptied during daylight hours, partway through the first day of each feeder full or feeder empty period, respectively (Figure 1). The specific time of day the feeder was either emptied or filled varied across replicates (see Table S3 for the specific fill/empty times). Earlier captive studies that have looked at the effect of food restriction on *T*_b_ have found evidence of low daytime *T*_b_ in Japanese quails (*Coturnix coturnix japonica*) during 3‐day fasts with 16 and 20 h of daylight, and upon repeated 3‐day fasts with 12 h of daylight (Laurila et al. 2005). We chose a longer period of food restriction as we were unable to fast individuals and anticipated that the effect of food removal would be buffered by chickadees exploiting caches initially when feeders were emptied (Cowie et al. 1981; Stevens and Krebs 1986; Brodin 1992), as well as by having access to natural food sources outside of caches. Stevens and Krebs (1986) found that in a related species, the Marsh tit (*Parus palustris*), attempts to retrieve seeds primarily occur within 12 daylight hours of the seeds being cached, and that all successful retrievals typically occur within ~24 h of daylight. In our system in winter, this would correspond to 2–3 days. Thus, the 6‐day window was expected to be sufficient for caches to be significantly reduced. Despite the fact that natural food was available throughout the study, we still anticipated that manipulating the availability of supplemental food would provide a meaningful manipulation of energy acquisition because even when natural food is available, supplemental feeders have been shown to increase survival by ~32% in black‐capped chickadees (Brittingham and Temple 1988). These differences in survival likely reflect differences in starvation risk, though they may also reflect differences in exposure to predators, as birds without supplemental food have to travel more to meet their daily energy intake (Roth and Vetter 2008). Consistent with this, chickadees in our study readily resumed intensive use of the thermal feeder once it was refilled. Mean daily feeder visits when the feeder was full (97.2 ± 42.1 visits/day/bird) were approximately equal to estimated mean daily energy requirements (~70 seeds at ~10°C, Lajoie et al. 2019), suggesting that the supplemental feeders represented an important food source. To allow for repeated measures of how individuals respond to experimentally manipulated food availability, we alternated between empty and full feeder periods for a total of six experimental replicates (see Table S3 for a breakdown of the experimental timeline).

Although the seven other feeders distributed in our study site were not equipped with an antenna and RFID reader capable of detecting the thermal tags, we synchronized the availability of supplemental food at all feeders to ensure that chickadees could not simply move to another feeder for access to supplemental food. Throughout the experiment, feeders were visited by observers every 3 days to retrieve data, change batteries, and fill or empty the feeder as needed (see Table S3). In this way, the level of disturbance at feeders was independent of the feeder treatment (full versus empty).

### 2.3 Data Processing and Selection

In total, we detected *N* = 24 birds using the feeder from 15 December 2023 to 24 February 2024 (*N* = 17 birds implanted in Fall 2023, *N* = 7 birds implanted in Fall 2022). We processed and filtered data from these 24 birds in three steps. First, we filtered *T*_sub_ data to ensure that patterns reported were not artifacts of erroneous measurements. As described in Hawkshaw et al. (2025), there were several instances in which a detection of an individual with a thermal PIT tag at the feeder did not have an associated *T*_sub_ recorded, either due to a failure to record a temperature or because the temperature read was outside the upper (50°C) or lower (25°C) limits of what the tags could measure (represented with a HH.H or LL.L message, respectively). In total, there were 234 detections that had no *T*_sub_ recorded out of a total of 132,645 detections (0.18%). Of these 234 detections, 14 were above the upper limit of the tags (0.01%) and 179 were below the lower limit of the tags (0.13%). There was one individual for which every detection at the feeder (*N* = 170 detections) was below the lower limit of the tags and thus, this individual was excluded from our analysis.

We also filtered out so‐called “spurious” *T*_sub_ detections, following Hawkshaw et al. (2025). Spurious *T*_sub_ detections were defined as detections that differed by > 1°C from the preceding and subsequent *T*_sub_ recording for the same individual, often within seconds of each other (see Text S1 for detailed filtering criteria). In total, 113 detections (0.09%) were labeled as spurious, which is similar to rates of spurious detections reported previously (0.17%, Hawkshaw et al. 2025). For detections that were labeled as “spurious” we retained the date and time of the detection but reported the *T*_sub_ value as NA. We acknowledge that our criteria for filtering spurious detections were subjective and have the potential to filter out true changes in *T*_b_. Therefore, we ran analyses both with and without the spurious detections in the dataset to confirm that these criteria did not influence our results, which they did not (results from data set including “spurious” detection are presented in Table S4).

During screening, we identified two individuals (IDs: 3D9.20D4817F31 and 3D9.20D481BE1) that had *T*_sub_ values that were consistently lower than the other individuals for several days in the study period (Figures S1–S23). We do not know what led to these individuals having consistently low *T*_sub_ values and whether they represent the real *T*_sub_ of the birds (e.g., due maintenance of low *T*_b_, poor health, or lead up to death) or if something happened to the tags (e.g., tag migration) that could account for the consistently lower *T*_sub_ readings. To confirm that inclusion of those individuals in our analyses did not have undue influence on our results, we ran all analyses on datasets with and without these individuals included. Generally, results from the dataset that excluded these two individuals were qualitatively similar to results from datasets that included them (results from data set including two outlier individuals is presented in Table S4). However, we found that exclusion of these individuals tended to improve the visual appearance of model residuals; thus, we report results from the analysis with these two individuals excluded in the main text.

As a second step in our data selection and filtering, we condensed repeated detections of the same bird into unique feeder visits. Our RFID antenna system was set up to scan PIT tags at 1 s intervals, which allowed for a single visit to the feeder to produce multiple lines of data. Previous work by Arteaga‐Torres et al. (2020) found that chickadees never return to the feeder within 12 s of their previous visit. Therefore, we condensed all sequential detections of an individual that occurred within 12 s of each other into the same feeder visit and reported the date and time of the first detection within that visit. For each feeder visit, we subsequently calculated the median *T*_sub_ of all detections within that visit (hereafter, “visit *T*_sub_”).

Finally, as we were interested in assessing whether chickadees adjust their use of daytime *T*_sub_ reductions in response food availability at the bird feeder, we excluded individuals that used the thermal RFID equipped feeder infrequently during our experiments. Birds that were detected infrequently at the thermal feeder did not rely on the thermal feeder as a primary source of food. If these birds were using other feeders at the study site, the food manipulation would still be equally strong for them, as treatments were synchronized across all feeders at the study site. However, these birds might also have been using other sources (e.g., caches, natural food, feeders outside of the study area), in which case the impact of the food manipulation would have been diluted for them. As we had no way to evaluate which of these scenarios was occurring for any bird with infrequent detections at the thermal feeder, we chose to restrict our analyses to birds for which we were confident the thermal feeder was an important food source. Our criteria for inclusion were as follows: (1) Once a bird was detected using the feeder when sunflower seeds were available, they needed to be present for all experimental replicates until either they were no longer detected at the feeder (i.e., due to mortality or tag loss) or the experiment ended. (2) During an experimental replicate, the bird needed to be detected for a minimum of three of the 5 days when the feeder was filled with sunflower seeds. These criteria resulted in the exclusion of an additional six birds, resulting in a sample size of *N* = 15 birds in the analyses presented in the main text (two outlier individuals excluded). Each of these 15 birds visited the feeder on average ≥ 58 times in a day. A previous study estimated that chickadees need to consume ~70 black‐oil sunflower seeds in a day to meet daily energy requirements of 65.5 kJ when the ambient temperature is circa −10°C (Lajoie et al. 2019). This suggests that the 15 birds included in our analyses were obtaining > 80% of their daily energy requirements from feeders when they were full.

### 2.4 Statistical Analyses

To evaluate *T*_sub_ responses to experimental manipulation of *ad libitum* food availability, we used data restricted to days in which the feeder was full or empty for the entire day (i.e., we excluded transition days). We constructed a linear mixed effect model with visit *T*_sub_ as the response variable. We included the following fixed effects in interaction with feeder status (full or empty) because each of these has previously been shown to influence daytime *T*_b_ in small birds: *T*_a_ (e.g., Laurila and Hohtola 2005; Lewden et al. 2014; Winder et al. 2020; Hawkshaw et al. 2025), daylength (e.g., Laurila et al. 2005; Hawkshaw et al. 2025), time of day (represented as hour in the day) (e.g., Lewden et al. 2014; Winder et al. 2020), and time of day^2^ (e.g., Winder et al. 2020). By modeling each of these predictors in interaction with feeder status (full or empty), we were able to evaluate whether their effects on *T*_sub_ differed depending on whether chickadees had access to supplemental food. We additionally included number of days since the feeder transitioned to full or empty to evaluate whether treatment effects changed over time. Individual ID and replicate number were included as random effects (intercepts) to account for the non‐independence of repeated measures from the same individuals and repeated measures from the same replicate. Days since the feeder transitioned and hourly *T*_a_ were left‐zeroed and daylength and time of day were centered so that the model intercept reflected estimated visit *T*_sub_ at noon (12:00 PM) on the first complete day the feeder was full within a replicate at the coldest hourly *T*_a_ (−34.0°C) and for the average daylength (8.56 h) experienced in the study. Each covariate was also standardized by dividing by two standard deviations (SD) such that estimates reflected the effect of changing each covariate by two SD to aid in comparing and interpretating the model estimates of these covariates (and their interactions) with feeder status (binary variable) as suggested by Gelman (2008). For days since transition this represented a change of 3.16 days, for hourly *T*_a_ a change of 16.49°C, for time of day a change of 6.63 h and for daylength a change of 1.96 h of daylight. We obtained hourly *T*_a_ data for the study period from the Edmonton International Airport weather station, 10 km SE of the study area from the Agriculture and Irrigation, Alberta Climate Information Service (https://www.acis.alberta.ca/acis/weather‐data‐viewer.jsp) and daylength data from the sunrise/sunset calculator from the National Research Council Canada (https://nrc.canada.ca/en/research‐development/products‐services/software‐applications/sun‐calculator/).

We performed all statistical analyses in the R statistical environment (v. 4.4.1, R Core Team 2024) and fit the linear mixed effects models using the “lmer” function in the R package “lme4” (Bates et al. 2015). For each model we assessed model fit by visually assessing plots of residuals. For each fixed effect in the linear mixed models, we calculated the mode and 95% credible interval (CrI) of the posterior distribution by generating 1000 simulations of the linear mixed model using the “sim” function from the R package “arm” (v. 1.14‐4, Gelman and Su 2024) and using the “posterior. mode” (R package “coda”, v. 0.19‐4.1) and “HPDinterval” (R package, “MCMCglmm”, v. 2.36) functions. We evaluated support for model effects following Marsman and Wagenmakers (2017), such that model estimates with 95% CrIs that did not overlap zero were interpreted as providing strong support for an effect while estimates with 95% CrIs that were centered on zero were interpreted as providing strong support for lack of an effect. For estimates with 95% CrIs that were not centered on zero but overlapped zero, we calculated the proportion of overlap (pr). For positive estimates we divided the number of estimates that were < 0 by the total number of estimates, while for negative estimates we divided the number of estimates that were > 0 by the total number of estimates. When the proportion of overlap was < 0.15, we interpreted this as moderate support for an effect in the reported direction, as this provides > 5 times greater support for interpretation of an effect in the reported direction compared to the opposite direction (i.e., 0.85/0.15 > 5) (Marsman and Wagenmakers 2017). Lastly, we estimated the adjusted repeatability (*r*) of visit *T*_sub_, using the “rpt” function in the R package “rptR” (v. 0.9.22, Stoffel et al. 2017).

## 3 Results

Throughout our experiment there were 17 chickadees that consistently used the thermal feeder when food was available (15 when excluding the two outlier individuals). Use of the feeder varied depending on the presence of food at the feeder with birds visiting the feeder more when it was full (*N* = 17 birds, *N* = 38, 968 total visits) as compared to when it was empty (*N* = 17 birds, *N* = 758 total visits). Additionally, there were 4 days where no chickadee was detected at the thermal feeder; 3 days when the feeder was empty (30 December, 12 January, 13 January) and 1 day when the feeder was refilled (14 January). When the feeder was full, the visit *T*_sub_ of chickadees was, on average, 42.2°C ± 1.6°C while when the feeder was empty visit *T*_sub_ was, on average, 42.1°C ± 1.3°C. With the two individual outlier individuals excluded, visit *T*_sub_ when the feeder was full was, on average, 42.5°C ± 0.6°C and 42.3°C ± 0.7°C when it was empty.

**FIGURE 2:**
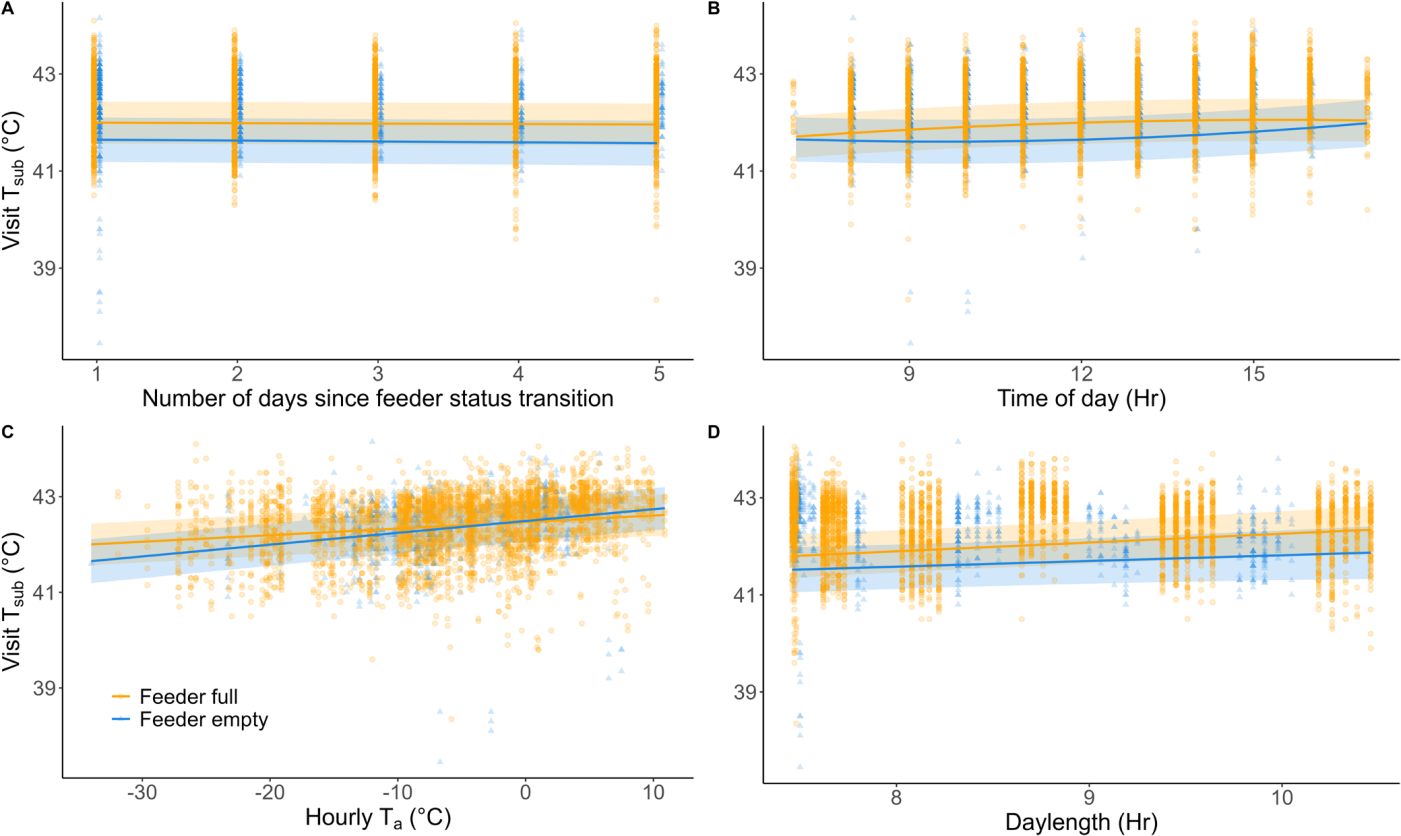
Effect of the availability of supplemental food at a bird feeder on visit *T*_sub_ in active black‐capped chickadees in relation to (A) number of days since the feeder status transitioned, (B) time of day, (C) hourly *T*_a_, and (D) daylength. In all panels linear trendlines with 95% CI (shaded areas) were generated using the “ggeffects” package (v. 1.7.0, Lüdecke 2018), and dots represent raw data points. In Panel (A) linear trendlines represent predicted values of visit *T*_sub_ when hourly *T*_a_ was at its minimum, and time of day and daylength were held at their mean. In Panel (B) linear trendlines represent predicted values of visit *T*_sub_ on the first full day since the feeder status transitioned when hourly *T*_a_ was at its minimum, and daylength was held at its mean. In Panel (C) linear trendlines represent predicted values of visit *T*_sub_ on the first full day since the feeder status transitioned when time of day and daylength were held at their mean. In Panel (D) linear trendlines represent predicted values of visit *T*_sub_ on the first full day since the feeder status transitioned when hourly *T*_a_ was at its minimum and time of day was held at its mean. For visualization purposes all raw data from when the feeder was empty was plotted, but only every 10th raw datapoint when the feeder was full was plotted.

There was a weak negative effect of the number of days since the feeder status transitioned on visit *T*_sub_, however, these effects did not differ with respect to whether the feeder was empty or full (Table 1 and Figure 2A). There was strong support that visit *T*_sub_ varied with time of day and that the non‐linear effect of time of day differed depending on feeder status (Table 1). On days when the feeder was full, *T*_sub_ slightly increased until around 3:30 PM, after which it decreased until the end of the foraging window, while on days when the feeder was empty, *T*_sub_ decreased until around 9:30 AM after which it increased until the end of the foraging window (Figure 2B). This resulted in chickadees between the two treatments having larger differences in *T*_sub_ in the middle of the day, with higher *T*_sub_ values occurring when supplemental food was available compared to when it was not (estimated difference: β = 0.4°C, 95% CI = 0.2°C–0.5°C on the first day of feeder transition, and at hourly *T*_a_ = −34.0°C and daylength set to the study average). However, *T*_sub_ was similar at the start and end of the foraging window regardless of feeder status. Overall, we found strong support that the visit *T*_sub_ of active chickadees was lower when hourly *T*_a_ was lower, and this effect was stronger when the feeder was empty compared to when it was full (Table 1). Across the full range of hourly *T*_a_ in our study (minimum = −34.0°C, maximum = 10.9°C), this corresponded to a change of β = 0.6°C (95% CI = 0.6°C–0.7°C) when the feeder was full, and β = 1.1°C (95% CI = 0.9°C–1.3°C) when the feeder was empty (estimated at average daylength and average time of day). As a result, the difference between the visit *T*_sub_ of chickadees when the feeder was empty compared to when it was full was largest at the lowest hourly *T*_a_ experienced during the study, with chickadees having warmer *T*_sub_ (β = 0.4°C, 95% CI = 0.2°C–0.5°C) when the feeder was full. This difference decreased as hourly *T*_a_ increased, and *T*_sub_ was slightly lower in food supplemented chickadees at the warmest *T*_a_ (10.9°C) in our study (β = −0.1°C, 95% CI = −0.2°C–0.0°C, Figure 2C). There was also strong support for a positive effect of daylength on visit *T*_sub_; the visit *T*_sub_ of chickadees tended to be higher on days with more hours of daylight, and this effect was greater when the feeder was full compared to when it was empty (Table 1 and Figure 2D). Across the full range of daylengths in our study (minimum = 7.46 h, maximum = 10.46 h), this corresponded to a change of β = 0.5°C (95% CI = 0.2°C–0.9°C) with food supplementation, and β = 0.4°C (95% CI = 0.0°C–0.7°C) without food supplementation (at hourly *T*_a_ = −34.0°C and daylength set to the study average). We also observed high among‐individual repeatability of visit *T*_sub_ (*r* = 0.66).

**TABLE 1:** Effect of supplemental food availability, hourly *T*_a_, daylength and time of day on daytime visit *T*_sub_ in active overwintering black‐capped chickadees (*N* = 37, 820 visits for *N* = 15 individuals). Models were run on a dataset excluding two individuals that had consistently low *T*_b_ for several days compared to other individuals in the study (IDs: 3D9.20D4817F31 and 3D9.20D481BE1), and spurious *T*_sub_ detections. Model effects presented are the mode of the posterior distribution with 95% CrI. The proportion of estimates overlapping zero (pr) is provided where applicable.

Fixed effect	β (95% CrI)
Intercept^a^	41.98 (41.59, 42.43)
Feeder. status: Empty	−0.33 (−0.49, −0.19)
Days since transition^b^	−0.03 (−0.05, −0.02)
Days since transition^b^ × Feeder. status: Empty	−0.03 (−0.10, 0.03) pr = 0.22
Hourly *T*_a_ ^c^	0.22 (0.21, 0.24)
Hourly *T*_a_ ^c^ × Feeder. status: Empty	0.17 (0.10, 0.25)
Daylength (h)^d^	0.32 (0.15, 0.55)
Daylength (h)^d^ × Feeder. status: Empty	−0.13 (−0.20, −0.06)
Time of day^e^	0.22 (0.21, 0.23)
Time of day^e^ × Feeder. status: Empty	0.00 (−0.09, 0.09)
Time of day^2^ ^e^	−0.22 (−0.24, −0.19)
Time of day^2^ ^e^ × Feeder. status: Empty	0.50 (0.29, 0.74)
**Random effect**	**α (95% CrI)**
Individual ID	0.47 (0.46, 0.47)
Replicate	0.07 (0.04, 0.17)
Residual	0.14 (0.14, 0.14)
**Repeatability** ^**f**^	***Adjusted r* (95% CrI)**
Individual ID	0.66 (0.49, 0.80)

^a^ Reference level for Feeder. status was set to “Full” such that intercepts were estimated when the feeder was full.

^b^ Days since transition was left‐zeroed and standardized such that intercepts were estimated for the first full day the supplemental feeder was full or empty and estimates represent the effect of 2 SD (3.16 days).

^c^ Hourly *T*_a_ was standardized and left‐zeroed such that intercepts were estimated at the coldest *T*_a_ (−34.0°C) and estimated effects represent as change in 2 SD (16.49°C).

^d^ Daylength was centered and standardized such that the intercepts were estimated at the mean daylength 8.56 h and estimated effects represent a changed 2 SD (1.96 h of daylength).

^e^ Time of day (h) was centred and standardized such that intercepts were estimated at noon (12:00 pm) and estimated effects represent a change in 2 SD (6.63 h).

^f^ Adjusted repeatability was calculated using the rptR package (Stoffel et al. 2017).

## 4 Discussion

We investigated whether experimental manipulations of supplemental food availability influenced daytime *T*_sub_ in active free‐living black‐capped chickadees in winter. As predicted, *T*_sub_ decreased with decreasing ambient temperature under both food treatments. This effect was larger when supplemental food was not available such that differences in *T*_sub_ were greatest when chickadees experienced the simultaneous challenge of low *T*_a_ and no access to supplemental food. We additionally found strong support that our experimental manipulations influenced the diurnal *T*_sub_ patterns, where under *ad libitum* food availability *T*_sub_ showed shallow increases until mid‐afternoon, and under natural food availability *T*_sub_ showed shallow decreases until mid‐morning after which it increased. Below we discuss the implications of these results for understanding how decreases in daytime *T*_b_ may be used as a strategy in small overwintering birds, in addition to limitations of our study and future directions.

We found that *T*_sub_ responses to our manipulations of supplemental food availability differed with environmental context. Chickadees generally had lower daytime *T*_sub_ when the hourly *T*_a_ was lower and, as predicted, chickadees responded more strongly when supplemental food was not available. At the lowest daytime hourly *T*_a_ experienced in the study (−34.0°C), chickadees had lower daytime *T*_sub_ when food was not available at bird feeders compared to when they had *ad libitum* access (estimated difference: 0.4°C, 95% CI = 0.2°C–0.5°C) but at milder *T*_a_, daytime *T*_sub_ was more similar between the two treatments (e.g., at *T*_a_ circa 11°C estimated difference = −0.1°C, 95% CI = −0.2°C to 0.0°C). This varying response to supplemental food availability based on ambient temperature suggests that chickadees may rely more heavily on low daytime *T*_b_ when the risk of energetic shortfall is particularly high. Similar to our findings, previous work on pigeons in outdoor aviaries demonstrated different effects of fasting under varying *T*_a_, where at temperatures around −25°C the difference in daytime *T*_b_ between pigeons with *ad libitum* access to food and pigeons on the first day of a fast was ~1.8°C, while at temperatures around 0°C the difference was only 0.4°C (extracted from figure 3 and 4 in Laurila and Hohtola 2005). Although the combined effect of hourly *T*_a_ and the food supplementation treatment in the present study is small (~0.4°C) compared to that observed in the study by Laurila and Hohtola (2005) (~1.8°C), a major difference between these studies is the extent to which the availability of food was manipulated. In our study, individuals always had access to naturally available food, while in Laurila and Hohtola (2005), individuals either had access to *ad libitum* food or were fasted. Given that in the absence of supplemental food, chickadees in our study still had access to natural food sources, including food caches, it is not surprising that the effect sizes we observed are smaller. Previous studies have shown that access to larger amounts of energy stores influence the maintenance of homeothermy (Hetem et al. 2016 and references therein) and can reduce torpor use (e.g., Humphries et al. 2003; though see Vuarin et al. (2013) for an example where increased energy stores lead to increased use of torpor). As such, the chickadees in our study, which had access to natural food, may not have needed to use substantial reductions in *T*_sub_ or may have been better able to maintain homeothermy compared to if they had been food restricted. Although the absolute magnitude of the decrease in *T*_sub_ observed in the present study was small, the estimated energy savings from these daytime reductions in *T*_sub_, assuming they reflect a strategic reduction in metabolic heat production, could be biologically important. A reduction in *T*_b_ of 0.4°C at an ambient temperature of −34.0°C could provide an energetic savings of ~3% to 5% (Range given 95% CI of T_sub_ reduction: 1.6%–6.0%), though the actual energy savings remains unclear given both the uncertainty in our estimated effect size and uncertainty in the parameters used to calculate energy savings (see Text S2 for further details). Thus, while it is conceivable that the small effect sizes reported here could have survival consequences when chickadees are faced with the simultaneous challenges of low food availability and low temperatures, the uncertainty surrounding the estimate makes the biological importance less clear. Measurement of metabolic rates would provide a more direct means of evaluating the true energetic implications of these modest changes in *T*_sub_.

We also explored whether diurnal patterns in *T*_sub_ differed with respect to the availability of supplemental food based on previous work that demonstrated midday peaks in bill temperature in great tits with *ad libitum* supplemental food, and midday drops in bill temperature under food restriction (Winder et al. 2020). Reductions in peripheral temperature arise via vasoconstriction to reduce heat loss (Tattersall et al. 2017), and can occur as instantaneous responses to food availability (Winder et al. 2020). However, we were interested in assessing whether *T*_sub_ might show similar patterns, albeit over longer time scales. We found strong support that diurnal patterns in *T*_sub_ changed in response to our manipulations of supplemental food availability as predicted. When chickadees had *ad libitum* access to supplemental food, they exhibited shallow increases in *T*_sub_ from the start of the foraging window till mid‐afternoon (~3:30 PM), after which it decreased until the end of the foraging window. In contrast, when the feeder was empty, chickadees exhibited a shallow decrease in *T*_sub_ from the start of the foraging window until around mid‐morning (~9:30 AM), after which it increased until the end of the foraging window. This observed change in diurnal *T*_sub_ patterns caused by our manipulations of supplemental food availability may indicate that during the feeder empty treatment, chickadees initially limit energy expenditure in the morning and later have to work harder to find food and attain sufficient energy stores before nightfall, thus leading to increases in *T*_sub_ toward the later part of the day. Such effects of increased workload on *T*_b_ have been found in marsh tits, which exhibit increased *T*_b_ in response to increased brood size (Nilsson and Nord 2018). It is also possible that during the feeder empty treatment chickadees relied more heavily on shallow torpor during the rest‐phase and, thus, did not need to forage as intensely in the morning as compared to when they had *ad libitum* access to the food in the bird feeder. Unfortunately, we were unable to obtain rest‐phase measures of *T*_sub_ and evaluate this possibility. However, at least in partial support of this idea, female great tits exhibit lower rest‐phase *T*_b_ when they do not have access to supplemental feeders (Nilsson et al. 2020). In contrast, as suggested by Winder et al. (2020) the mid‐afternoon peaks in *T*_sub_ when supplemental food was available *ad libitum* may result from heat produced via digestion and/or activity during the morning and early afternoon. In captive pigeons, diurnal patterns in *T*_b_ have been shown to align with feeding and activity schedules (Rashotte et al. 1995). Although our results for the effect of manipulations of food availability on diurnal patterns of *T*_sub_ are aligned with previously reported patterns in bill temperatures for great tits (Winder et al. 2020), the estimated effect size in the present study was again relatively small (maximum difference estimated at midday 0.4°C in Figure 2C) compared to the differences in bill temperature reported by Winder et al. (2020) (~4.2°C, data extracted from figure 4 in Winder et al. 2020). The smaller effect size observed in the present study may reflect differences in thermoregulation at different areas of the body (periphery, Winder et al. 2020 vs. *T*_sub_ in the present study).

Although we did not have strong a priori predictions about how daylength might mediate the effect of our food manipulation, previously work has shown that *T*_b_ varies with daylength (Laurila et al. 2005; Dawson 2017; Appenroth et al. 2021; Hawkshaw et al. 2025). Therefore, we also evaluated whether the effect of supplemental food availability was dependent on daylength. We found that daytime *T*_sub_ increased with increasing daylength and this effect was stronger when chickadees had access to supplemental food at feeders compared to when supplemental food was not provided. However, the estimated effects (0.4°C, 95% CI 0.0°C–0.7°C and 0.5°C, 95% CI 0.2°C–0.9°C change across the full range of daylengths when the feeder was empty and full, respectively) were relatively small. The positive effect of daylight hours of *T*_sub_ we report here may be the result of chickadees being more active and thus producing more heat via activity on longer days. However, increased activity, at least as inferred by the time elapsed between successive feeder visits, has previously been shown to be associated with lower daytime *T*_sub_ in chickadees (Hawkshaw et al. 2025). Interestingly, the overall effect of daylength we observed, contrasts with that previously reported in chickadees (Hawkshaw et al. 2025), quails (Laurila et al. 2005), starlings ( *Sturnus vulgaris* , Dawson 2017), and Svalbard rock ptarmigan ( *Lagopus muta hyperborea* , Appenroth et al. 2021), which have found lower daytime *T*_b_ in response to longer daylengths. Conditions across these studies and the present study have varied with respect to the experimental setting (lab vs. field), and type of food availability manipulation (fasted/*ad libitum* vs. continuous access to supplemental food/intermittent access to supplemental food), so it is unclear which of these factors may explain our contrasting result for effects of daylength on *T*_sub_.

We found strong support for various effects of manipulations of supplemental food on *T*_sub_ in free‐living chickadees; however, the effect sizes were relatively small compared to other studies reporting effects of food restrictions on body temperature (e.g., Chaplin et al. 1984; Reinertsen and Haftorn 1986; Graf et al. 1989; Rashotte et al. 1998; Laurila et al. 2005; Nilsson et al. 2020). We suggest that the differences in the extent of the food manipulation treatments between the present study and those in some earlier works likely account, at least in part, for the modest effect sizes observed here (e.g., compared to Graf et al. 1989; Rashotte et al. 1998; Laurila et al. 2005). Additionally, the nature of our *T*_b_ measurements resulted in us only recording measures of *T*_sub_ while birds were active and had just engaged in flight in order to visit the feeder, while earlier studies reported reduction in rest‐phase body temperature in response to food manipulations (Chaplin et al. 1984; Reinertsen and Haftorn 1986; Rashotte et al. 1998; Laurila et al. 2005; Nilsson et al. 2020). As such, our measures of daytime *T*_sub_ include any heat produced during movements to the feeder, as well as other underlying processes (e.g., digestion), both of which may have obscured changes in *T*_sub_. It is possible that by engaging in flight, individuals may have rewarmed to similar levels before visiting the feeder. Contrary to this idea, work from Cooper and Sonsthagen (2007) indicates that measures of *T*_b_ and heat production in perching versus active chickadees in captivity are similar. However, movements of individuals in the former study would have been more restricted compared to individuals in our study that were free‐ranging, and therefore, their findings may not translate. Additionally, previous work in chickadees has shown that shorter‐time intervals between feeder visits (a proxy for increased activity) are associated with lower *T*_sub_ (Hawkshaw et al. 2025). It is also possible that the chickadees were all equally active during both treatment periods trying to obtain food (at the feeder when food was available and at alternative natural food sources when the feeder was empty), and thus, our measure of *T*_b_ were capturing similar levels of activity and heat generation. However, combined measures of *T*_b_ and activity from individuals throughout their active period would be needed to confirm how changes in activity are associated with changes in *T*_b_. Further, as we were only able to measure *T*_sub_ in active chickadees when present at the feeder, our data do not allow us to assess whether nocturnal *T*_b_ or daytime *T*_b_ at other points in the active phase changed. It is possible that chickadees exhibited stronger adjustments of their nocturnal *T*_sub_ or while resting between bouts of foraging compared to the observed changes in daytime changes in active *T*_sub_. Previous work on pigeons in captive settings has found that when food deprived, changes in nocturnal *T*_b_ are greater than daytime *T*_b_ (Graf et al. 1989; Rashotte et al. 1998) or that decreases in daytime *T*_b_ only occur in extreme cold (Laurila and Hohtola 2005). Together, both our results and the results of these earlier studies (Graf et al. 1989; Rashotte et al. 1998; Laurila and Hohtola 2005) suggest that the constraints on reductions in body temperature are greater during the active phase compared to the rest phase. To fully grasp the extent of energy savings that might be induced by defending lower *T*_b_ in response to food manipulations and low ambient temperatures, estimates of changes in nocturnal *T*_b_ and changes in *T*_b_ at other points during the day are needed.

These limitations notwithstanding, our study provides strong support that experimental manipulations of supplemental food availability in free‐living chickadees elicit changes in *T*_sub_. Although the effect sizes observed are small, they are consistently in the direction predicted by theory. To our knowledge, this study is one of the few to experimentally investigate the role of food availability on daytime *T*_b_ in an avian species under natural conditions. Despite the fact that chickadees in our study still had access to natural food sources during the food removal treatment, changes in diurnal patterns of *T*_sub_ and responses to *T*_a_ were qualitatively similar to patterns reported in previous studies that were able to completely restrict access to food and/or obtained different measures of *T*_b_ (Laurila and Hohtola 2005; Winder et al. 2020). The smaller magnitude of food limitation we were able to implement likely had correspondingly smaller effects on *T*_sub_, and daytime reductions in *T*_sub_ might be expected to be shallower than rest‐phase reductions in *T*_sub_. While this study marks an important first step toward understanding how access to a reliable food source in winter may shape energy management in small wintering birds, future studies that obtain measures of *T*_b_ outside of when individuals are actively foraging are needed to provide a more complete understanding of the effect that food availability has on the use of adjustments in *T*_b_ as an energy management strategy.

## Author Contributions

**Deborah M. Hawkshaw:** conceptualization (equal), data curation (lead), formal analysis (lead), funding acquisition (supporting), investigation (equal), methodology (lead), validation (lead), visualization (lead), writing – original draft (lead), writing – review and editing (equal). **Kimberley J. Mathot:** conceptualization (equal), funding acquisition (lead), investigation (equal), methodology (supporting), project administration (lead), supervision (lead), validation (equal), writing – original draft (supporting), writing – review and editing (equal).

## Funding

D.M.H.'s graduate program was funded by an NSERC Canada Graduate Scholarship ‐Doctoral. This study was supported by an Alberta Conservation Association Grant in Biodiversity awarded to D.M.H. as well as by an NSERC Discovery grant (RGPIN‐2018‐04358) and Canadian Research Chair research funds awarded to K.J.M.

## Ethics Statement

All work was approved by and conducted in accordance with the University of Alberta Animal Care and Use Committee (AUP00002210), banding permits from the Bird Banding Office in Canada (Permits no. 10936 and 10936A), and an Alberta Research permit (No. 23‐016 and No. 24‐013).

## Conflicts of Interests

The authors declare no conflicts of interest.

## Supporting information


**Table S1:** Mean absolute temperature deviation of 63 temperature‐sensing passive integrated transponder (PIT) tags from a water bath at different test temperatures (range 25°C–46°C). Note, although only 21 tags were used in the study, we present data on the larger set of tags that were tested as this provides higher precision for estimating mean absolute deviations across temperatures. Test temperatures between 33°C and 43°C represent temperatures within the manufacturer's specified range for the thermal PIT tags, while temperatures < 33°C and > 43°C are outside the manufacturers specified range. The water bath used for testing was a Fisher Scientific IsoTemp GPD10 (model no. FSGPD10) which was calibrated using a mercury glass thermometer prior to testing. The accuracy of the water bath was ±0.2°C, while the accuracy of the tags within the manufacturer specified range for the thermal tags was ±0.5°C. To calculate the absolute temperature deviation, for each tag at each test temperature, the mean tag and water bath temperature was calculated. The absolute difference between the mean tag temperature and water bath temperature was taken. For each test temperature the mean of the absolute temperature deviations (across all tags) was then calculated.
**Table S2:** Mean absolute temperature deviation for each implanted temperature‐sensing passive integrated transponder (PIT) tags from a water bath across the different test temperatures (25°C–46°C). Test temperatures between 33°C and 43°C represent temperatures within the manufacturer's specified range for the thermal PIT tags, while temperatures < 33°C and > 43°C are outside the manufacturers specified range. The water bath used for testing was a Fisher Scientific IsoTemp GPD10 (model no. FSGPD10) which was calibrated using a mercury glass thermometer prior to testing. The accuracy of the water bath was ±0.2°C, while the accuracy of the tags was ±0.5°C within the manufacturer specified range. To calculate the absolute temperature deviation, for each tag at each test temperature, the mean tag and water bath temperature was calculated. The absolute difference between the mean tag temperature and water bath temperature was taken. For each thermal tag, the mean of the absolute temperature deviations (across all test temperatures) was then calculated. Thermal tags are ordered alphanumerically.
**Table S3:** Date, replicate number, and status of the thermal feeder during the supplemental food manipulation experiment (from 15 December 2023 to 24 February 2024). Every 3 days the feeder was visited to retrieve data, replace batteries, and refill or empty the feeder as needed as well as to equalize the amount of disturbance occurring at each of the feeders in the study site regardless of feeder status.
**Table S4:** Effects of supplemental food availability, hourly ambient *T*_a_, daylength and time of day on visit *T*_sub_ in active chickadees in winter. Models were run on datasets that applied different data filtering to ensure that subjective data filtering decisions did not unduly influence model inferences. Model effects presented are the mode of the posterior distribution with 95% CrI, while the adjusted repeatability estimates are the point estimates and 95% CrI. The proportion of estimates overlapping zero (pr) are provided where applicable.
**Figure S1:** Daily plots of subcutaneous body temperatures (*T*_sub_) in relation to time of day for individual 3D9.20D4A81BE1 while they were detected at the thermal feeder during the food manipulation experiment. Black dots represent *T*_sub_ detections that were not identified as spurious while red dots represent *T*_sub_ detections that were identified as spurious.
**Figure S2:** Daily plots of subcutaneous body temperatures (*T*_sub_) in relation to time of day for individual 3D9.20D4A81FF7 while they were detected at the thermal feeder during the food manipulation experiment. Black dots represent *T*_sub_ detections that were not identified as spurious while red dots represent *T*_sub_ detections that were identified as spurious.
**Figure S3:** Daily plots of subcutaneous body temperatures (*T*_sub_) in relation to time of day for individual 3D9.20D4A565FA while they were detected at the thermal feeder during the food manipulation experiment. Black dots represent *T*_sub_ detections that were not identified as spurious while red dots represent *T*_sub_ detections that were identified as spurious.
**Figure S4:** Daily plots of subcutaneous body temperatures (*T*_sub_) in relation to time of day for individual 3D9.20D4A5643E while they were detected at the thermal feeder during the food manipulation experiment. Black dots represent *T*_sub_ detections that were not identified as spurious while red dots represent *T*_sub_ detections that were identified as spurious.
**Figure S5:** Daily plots of subcutaneous body temperatures (*T*_sub_) in relation to time of day for individual 3D9.20D4A5646A while they were detected at the thermal feeder during the food manipulation experiment. Black dots represent *T*_sub_ detections that were not identified as spurious while red dots represent *T*_sub_ detections that were identified as spurious.
**Figure S6:** Daily plots of subcutaneous body temperatures (*T*_sub_) in relation to time of day for individual 3D9.20D45667A while they were detected at the thermal feeder during the food manipulation experiment. Black dots represent *T*_sub_ detections that were not identified as spurious while red dots represent *T*_sub_ detections that were identified as spurious.
**Figure S7:** Daily plots of subcutaneous body temperatures (*T*_sub_) in relation to time of day for individual 3D9.20D4A5671F while they were detected at the thermal feeder during the food manipulation experiment. Black dots represent *T*_sub_ detections that were not identified as spurious while red dots represent *T*_sub_ detections that were identified as spurious.
**Figure S8:** Daily plots of subcutaneous body temperatures (*T*_sub_) in relation to time of day for individual 3D9.20D4A8213D while they were detected at the thermal feeder during the food manipulation experiment. Black dots represent *T*_sub_ detections that were not identified as spurious while red dots represent *T*_sub_ detections that were identified as spurious.
**Figure S9:** Daily plots of subcutaneous body temperatures (*T*_sub_) in relation to time of day for individual 3D9.20D4A56413 while they were detected at the thermal feeder during the food manipulation experiment. Black dots represent *T*_sub_ detections that were not identified as spurious while red dots represent *T*_sub_ detections that were identified as spurious.
**Figure S10:** Daily plots of subcutaneous body temperatures (*T*_sub_) in relation to time of day for individual 3D9.20D4A56459 while they were detected at the thermal feeder during the food manipulation experiment. Black dots represent *T*_sub_ detections that were not identified as spurious while red dots represent *T*_sub_ detections that were identified as spurious. Note: no detections were identified as spurious.
**Figure S11:** Daily plots of subcutaneous body temperatures (*T*_sub_) in relation to time of day for individual 3D9.20D4A56502 while they were detected at the thermal feeder during the food manipulation experiment. Black dots represent *T*_sub_ detections that were not identified as spurious while red dots represent *T*_sub_ detections that were identified as spurious.
**Figure S12:** Daily plots of subcutaneous body temperatures (*T*_sub_) in relation to time of day for individual 3D9.20D4A56520 while they were detected at the thermal feeder during the food manipulation experiment. Black dots represent *T*_sub_ detections that were not identified as spurious while red dots represent *T*_sub_ detections that were identified as spurious. Note: no detections were identified as spurious.
**Figure S13:** Daily plots of subcutaneous body temperatures (*T*_sub_) in relation to time of day for individual 3D9.20D4A56555 while they were detected at the thermal feeder during the food manipulation experiment. Black dots represent *T*_sub_ detections that were not identified as spurious while red dots represent *T*_sub_ detections that were identified as spurious.
**Figure S14:** Daily plots of subcutaneous body temperatures (*T*_sub_) in relation to time of day for individual 3D9.20D4A56738 while they were detected at the thermal feeder during the food manipulation experiment. Black dots represent *T*_sub_ detections that were not identified as spurious while red dots represent *T*_sub_ detections that were identified as spurious.
**Figure S15:** Daily plots of subcutaneous body temperatures (*T*_sub_) in relation to time of day for individual 3D9.20D4A82287 while they were detected at the thermal feeder during the food manipulation experiment. Black dots represent *T*_sub_ detections that were not identified as spurious while red dots represent *T*_sub_ detections that were identified as spurious.
**Figure S16:** Daily plots of subcutaneous body temperatures (*T*_sub_) in relation to time of day for individual 3D9.20D4A82296 while they were detected at the thermal feeder the food manipulation experiment. Black dots represent *T*_sub_ detections that were not identified as spurious while red dots represent *T*_sub_ detections that were identified as spurious. Note: no detections were identified as spurious.
**Figure S17:** Daily plots of subcutaneous body temperatures (*T*_sub_) in relation to time of day for individual 3D9.20D4817F1D while they were detected at the thermal feeder during the food manipulation experiment. Black dots represent *T*_sub_ detections that were not identified as spurious while red dots represent *T*_sub_ detections that were identified as spurious.
**Figure S18:** Daily plots of subcutaneous body temperatures (*T*_sub_) in relation to time of day for individual 3D9.20D4817F1E while they were detected at the thermal feeder during the food manipulation experiment. Black dots represent *T*_sub_ detections that were not identified as spurious while red dots represent *T*_sub_ detections that were identified as spurious.
**Figure S19:** Daily plots of subcutaneous body temperatures (*T*_sub_) in relation to time of day for individual 3D9.20D4817F25 while they were detected at the thermal feeder during the food manipulation experiment. Black dots represent *T*_sub_ detections that were not identified as spurious while red dots represent *T*_sub_ detections that were identified as spurious.
**Figure S20:** Daily plots of subcutaneous body temperatures (*T*_sub_) in relation to time of day for individual 3D9.20D4817F26 while they were detected at the thermal feeder during the food manipulation experiment. Black dots represent *T*_sub_ detections that were not identified as spurious while red dots represent *T*_sub_ detections that were identified as spurious.
**Figure S21:** Daily plots of subcutaneous body temperatures (*T*_sub_) in relation to time of day for individual 3D9.20D4817F28 while they were detected at the thermal feeder during the food manipulation experiment. Black dots represent *T*_sub_ detections that were not identified as spurious while red dots represent *T*_sub_ detections that were identified as spurious.
**Figure S22:** Daily plots of subcutaneous body temperatures (*T*_sub_) in relation to time of day for individual 3D9.20D4817F30 while they were detected at the thermal feeder during the food manipulation experiment. Black dots represent *T*_sub_ detections that were not identified as spurious while red dots represent *T*_sub_ detections that were identified as spurious.
**Figure S23:** Daily plots of subcutaneous body temperatures (*T*_sub_) in relation to time of day for individual 3D9.20D4817F31 while they were detected at the thermal feeder during the food manipulation experiment. Black dots represent *T*_sub_ detections that were not identified as spurious while red dots represent *T*_sub_ detections that were identified as spurious.

## Data Availability

All data and code for reproducible analyses are available at the OSF repository https://doi.org/10.17605/OSF.IO/JMQXU.
