# Supplementary material for: Evidence of Small Changes in Daytime Body Temperature in Active Black‐Capped Chickadees in Response to Supplemental Food Availability
Source: Ecol Evol. 2026 Jul 5;16(7):e73946. doi: 10.1002/ece3.73946 (PMC13333254; doi:10.1002/ece3.73946)
Supplement: Supplementary file 1 — Table S1: Mean absolute temperature deviation of 63 temperature‐sensing passive integrated transponder (PIT) tags from a water bath at different test temperatures (range 25°C–46°C). Note, although only 21 tags were used in the study, we present data on the larger set of tags that were tested as this provides higher precision for estimating mean absolute deviations across temperatures. Test temperatures between 33°C and 43°C represent temperatures within the manufacturer's specified range for the thermal PIT tags, while temperatures < 33°C and > 43°C are outside the manufacturers specified range. The water bath used for testing was a Fisher Scientific IsoTemp GPD10 (model no. FSGPD10) which was calibrated using a mercury glass thermometer prior to testing. The accuracy of the water bath was ±0.2°C, while the accuracy of the tags within the manufacturer specified range for the thermal tags was ±0.5°C. To calculate the absolute temperature deviation, for each tag at each test temperature, the mean tag and water bath temperature was calculated. The absolute difference between the mean tag temperature and water bath temperature was taken. For each test temperature the mean of the absolute temperature deviations (across all tags) was then calculated. Table S2: Mean absolute temperature deviation for each implanted temperature‐sensing passive integrated transponder (PIT) tags from a water bath across the different test temperatures (25°C–46°C). Test temperatures between 33°C and 43°C represent temperatures within the manufacturer's specified range for the thermal PIT tags, while temperatures < 33°C and > 43°C are outside the manufacturers specified range. The water bath used for testing was a Fisher Scientific IsoTemp GPD10 (model no. FSGPD10) which was calibrated using a mercury glass thermometer prior to testing. The accuracy of the water bath was ±0.2°C, while the accuracy of the tags was ±0.5°C within the manufacturer specified range. To calculate the absolute temperat [file ECE3-16-e73946-s001.docx]

**Electronic Supplementary Material for: Evidence of small changes in daytime body temperature in active black-capped chickadees in response to supplemental food availability**

Deborah M. Hawkshaw and Kimberley J. Mathot

**Table of Contents**

[**Supplemental Text S1.** Filtering “spurious” temperature detections 4](#_Toc230695050)

[**Supplementary Text S2**. Calculation of energy savings for observed changes in T_sub_ 4](#_Toc230695051)

[**Table S1.** Mean absolute temperature deviation of 63 temperature-sensing passive integrated transponder (PIT) tags from a water bath at different test temperatures (range 25-46 ℃). 7](#_Toc230695052)

[**Table S2.** Mean absolute temperature deviation for each implanted temperature-sensing passive integrated transponder (PIT) tags from a water bath across the different test temperatures (25-46 ℃). 8](#_Toc230695053)

[**Table S3.** Date, replicate number, and status of the thermal feeder during the supplemental food manipulation experiment (from 15 December 2023 to 24 February 2024). 9](#_Toc230695054)

[**Table S4.** Effects of supplemental food availability, hourly ambient T_a_, daylength and time of day on visit T_sub_ in active chickadees in winter. 11](#_Toc230695055)

[**Figure S1.** Daily plots of subcutaneous body temperatures (T _sub_) in relation to time of day for individual 3D9.20D4A81BE1 while they were detected at the thermal feeder during the food manipulation experiment. Black dots represent T _sub_ detections that were not identified as spurious while red dots represent T_sub_ detections that were identified as spurious. 13](#_Toc230695056)

[**Figure S2.** Daily plots of subcutaneous body temperatures (T _sub_) in relation to time of day for individual 3D9.20D4A81FF7 while they were detected at the thermal feeder during the food manipulation experiment. 14](#_Toc230695057)

[**Figure S3.** Daily plots of subcutaneous body temperatures (T _sub_) in relation to time of day for individual 3D9.20D4A565FA while they were detected at the thermal feeder during the food manipulation experiment. 15](#_Toc230695058)

[**Figure S4.** Daily plots of subcutaneous body temperatures (T _sub_) in relation to time of day for individual 3D9.20D4A5643E while they were detected at the thermal feeder during the food manipulation experiment. 16](#_Toc230695059)

[**Figure S5.** Daily plots of subcutaneous body temperatures (T _sub_) in relation to time of day for individual 3D9.20D4A5646A while they were detected at the thermal feeder during the food manipulation experiment. 17](#_Toc230695060)

[**Figure S6.** Daily plots of subcutaneous body temperatures (T _sub_) in relation to time of day for individual 3D9.20D45667A while they were detected at the thermal feeder during the food manipulation experiment. 18](#_Toc230695061)

[**Figure S7.** Daily plots of subcutaneous body temperatures (T _sub_) in relation to time of day for individual 3D9.20D4A5671F while they were detected at the thermal feeder during the food manipulation experiment. 19](#_Toc230695062)

[**Figure S8.** Daily plots of subcutaneous body temperatures (T _sub_) in relation to time of day for individual 3D9.20D4A8213D while they were detected at the thermal feeder during the food manipulation experiment. 20](#_Toc230695063)

[**Figure S9.** Daily plots of subcutaneous body temperatures (T _sub_) in relation to time of day for individual 3D9.20D4A56413 while they were detected at the thermal feeder during the food manipulation experiment. 21](#_Toc230695064)

[**Figure S10.** Daily plots of subcutaneous body temperatures (T _sub_) in relation to time of day for individual 3D9.20D4A56459 while they were detected at the thermal feeder during the food manipulation experiment. 22](#_Toc230695065)

[**Figure S11.** Daily plots of subcutaneous body temperatures (T _sub_) in relation to time of day for individual 3D9.20D4A56502 while they were detected at the thermal feeder during the food manipulation experiment. 23](#_Toc230695066)

[**Figure S12.** Daily plots of subcutaneous body temperatures (T _sub_) in relation to time of day for individual 3D9.20D4A56520 while they were detected at the thermal feeder during the food manipulation experiment. 24](#_Toc230695067)

[**Figure S13.** Daily plots of subcutaneous body temperatures (T _sub_) in relation to time of day for individual 3D9.20D4A56555 while they were detected at the thermal feeder during the food manipulation experiment. 25](#_Toc230695068)

[**Figure S14.** Daily plots of subcutaneous body temperatures (T _sub_) in relation to time of day for individual 3D9.20D4A56738 while they were detected at the thermal feeder during the food manipulation experiment. 26](#_Toc230695069)

[**Figure S15.** Daily plots of subcutaneous body temperatures (T _sub_) in relation to time of day for individual 3D9.20D4A82287 while they were detected at the thermal feeder during the food manipulation experiment. 27](#_Toc230695070)

[**Figure S16.** Daily plots of subcutaneous body temperatures (T _sub_) in relation to time of day for individual 3D9.20D4A82296 while they were detected at the thermal feeder the food manipulation experiment. 28](#_Toc230695071)

[**Figure S17.** Daily plots of subcutaneous body temperatures (T _sub_) in relation to time of day for individual 3D9.20D4817F1D while they were detected at the thermal feeder during the food manipulation experiment. 29](#_Toc230695072)

[**Figure S18.** Daily plots of subcutaneous body temperatures (T _sub_) in relation to time of day for individual 3D9.20D4817F1E while they were detected at the thermal feeder during the food manipulation experiment. 30](#_Toc230695073)

[**Figure S19.** Daily plots of subcutaneous body temperatures (T _sub_) in relation to time of day for individual 3D9.20D4817F25 while they were detected at the thermal feeder during the food manipulation experiment. 31](#_Toc230695074)

[**Figure S20.** Daily plots of subcutaneous body temperatures (T _sub_) in relation to time of day for individual 3D9.20D4817F26 while they were detected at the thermal feeder during the food manipulation experiment. 32](#_Toc230695075)

[**Figure S21.** Daily plots of subcutaneous body temperatures (T _sub_) in relation to time of day for individual 3D9.20D4817F28 while they were detected at the thermal feeder during the food manipulation experiment. 33](#_Toc230695076)

[**Figure S22.** Daily plots of subcutaneous body temperatures (T _sub_) in relation to time of day for individual 3D9.20D4817F30 while they were detected at the thermal feeder during the food manipulation experiment. 34](#_Toc230695077)

[**Figure S23.** Daily plots of subcutaneous body temperatures (T _sub_) in relation to time of day for individual 3D9.20D4817F31 while they were detected at the thermal feeder during the food manipulation experiment. 35](#_Toc230695078)

[**Supplementary References** 36](#_Toc230695079)

# **Supplemental Text S1.** Filtering “spurious” temperature detections

We filtered out so-called “spurious” T_sub_ detections from our RFID data following Hawkshaw et al. (2025). Spurious T_sub_ detections were defined as detections that differed by >1 ℃ from both the preceding and subsequent T_sub_ recording for the same individual and may have reflected read errors. To identify and filter out spurious detections, we applied the following three criteria. 1) A detection was flagged as spurious if, within a given day, the absolute change in T_sub_ between that detection and the previous detection with T_sub_ recorded was >1 ℃ and the absolute change in T_sub_ between that detection and the subsequent detection with T_sub_ recorded was >1 ℃. 2) For first detection of a day, the detection was considered spurious if the absolute change in temperature between that detection and the second detection of the day with an associated T_sub_ was >1 ℃ and the absolute change in temperature between the second and third detections of the day with temperature recorded was < 1 ℃. 3) For the last detection of a day, the detection was considered spurious if the absolute change in T_sub_ between that detection and the second last detection with T_sub_ recorded was >1 ℃ and the absolute change in T_sub_ between the third last and second last detection was < 1 ℃. We then visually assessed plots of T_sub_ vs Time for an individual for each day they visited the feeder to confirm that this filter method captured detections that were visually identifiable as spurious, which it did (ESM Figures S1-23).

# **Supplementary Text S2**. Calculation of energy savings for observed changes in T_sub_

We estimated the potential energy savings associated with defending a lower T_sub_ for black-capped chickadees (*Poecile atricapillus*). We specifically calculated how much energy savings would result from the observed reductions in T_sub_ (0.4 ^o^C) in the present study under the ambient conditions of our study (e.g., ambient temperatures -34.0 ^o^C). Defending a lower body temperature (T_b_) relative to the euthermic body temperature (T_euthermic_) results in energy savings in two ways. First, it reduces heat loss to the environment (Scholander et al., 1950) (Equation 1). Second, it reduces the metabolic demands of colder tissues (Chaui-Berlinck et al., 2002) (Equation 2).

Equation 1: $\Delta M=C \times\left( T_{euthermic}-T_{b} \right)$

Equation 2: $\Delta M=C \times\left( T_{euthermic}-T_{a} \right)\times(1-Q_{10}^{-\frac{T_{euthermic}- T_{b}}{10}})$

Where ΔM = change in metabolic rate. Descriptions of other equation parameters and the values used in calculations are listed below.

| Parameter | Description | Parameter value used in calculation | Source |
| --- | --- | --- | --- |
| C | Whole body thermal conductance, assuming average body mass of 11g. | 0.0132 W ^o^C^-1^ | Cooper and Swanson (1994) |
| T_euthermic_ | Body temperature of a euthermic chickadee | 42 ^o^C | Chaplin (1974), Chaplin (1976) |
| T_b_ | Body temperature of a chickadee with the observed 0.4 ℃ decreased in T_sub_ | 41.6 ^o^C | Present study |
| T_a_ | Ambient temperature. Value used reflects minimum temperatures observed during study | -34 ^o^C | Present study |
| Q_10_ | Coefficient indicating increases in biological or chemical reaction rates per 10 ^o^C increase in temperature. Unitless. | 2-3 | Schmidt-Nielsen (1997) |

Summing calculated values from Equation 1 and Equation 2, the estimated energy savings for magnitude of ΔT_sub_ decreases observed in the present study are between 0.033 to 0.048 W (assuming Q_10_ equals 2 or 3, respectively). Given the estimated metabolic rate for a euthermic chickadee at -34 ^o^C is 1.003 W (calculated as C × (T_euthermic_ - T_a_), the observed ΔT_sub_ in the present study equates to energy savings of ~ 3.3 to 4.8 %.

However, when considering the uncertainty in the estimated effect size (95% CI = 0.2 ^o^C to 0.5 ^o^C), the range of potential energy savings could be as low as 1.6% or as high as 6.0%. Assessing the biological importance of the observed changes in T_sub_ are further complicated by the fact that conductance and metabolic rate parameters used here are treated as fixed values, but in reality, also have associated uncertainty. Thus, these estimates are not intended to be interpreted as measured energy savings (they are not), but rather, as illustrative that even small changes in T_sub_ can generate meaningful energy savings if they arise via decreased metabolic heat production.

**Table S1.** Mean absolute temperature deviation of 63 temperature-sensing passive integrated transponder (PIT) tags from a water bath at different test temperatures (range 25-46 ℃). Note, although only 21 tags were used in the study, we present data on the larger set of tags that were tested as this provides higher precision for estimating mean absolute deviations across temperatures. Test temperatures between 33-43 ℃ represent temperatures within the manufacturer’s specified range for the thermal PIT tags, while temperatures <33 ℃ and >43 ℃ are outside the manufacturers specified range. The water bath used for testing was a Fisher Scientific IsoTemp GPD10 (model no. FSGPD10) which was calibrated using a mercury glass thermometer prior to testing. The accuracy of the water bath was ±0.2 ℃, while the accuracy of the tags within the manufacturer specified range for the thermal tags was ±0.5 ℃. To calculate the absolute temperature deviation, for each tag at each test temperature, the mean tag and water bath temperature was calculated. The absolute difference between the mean tag temperature and water bath temperature was taken. For each test temperature the mean of the absolute temperature deviations (across all tags) was then calculated.

| **Test temperature (℃)** | **Mean absolute temperature deviation from waterbath (℃)** |
| --- | --- |
| 25 | 0.3 |
| 26 | 0.3 |
| 27 | 0.4 |
| 28 | 0.5 |
| 29 | 0.5 |
| 30 | 0.5 |
| 31 | 0.5 |
| 32 | 0.4 |
| 33 | 0.5 |
| 34 | 0.5 |
| 35 | 0.4 |
| 36 | 0.5 |
| 37 | 0.5 |
| 38 | 0.3 |
| 39 | 0.3 |
| 40 | 0.2 |
| 43 | 0.1 |
| 44 | 0.2 |
| 45 | 0.2 |
| 46 | 0.2 |

**Table S2.** Mean absolute temperature deviation for each implanted temperature-sensing passive integrated transponder (PIT) tags from a water bath across the different test temperatures (25-46 ℃). Test temperatures between 33-43 ℃ represent temperatures within the manufacturer’s specified range for the thermal PIT tags, while temperatures <33 ℃ and >43 ℃ are outside the manufacturers specified range. The water bath used for testing was a Fisher Scientific IsoTemp GPD10 (model no. FSGPD10) which was calibrated using a mercury glass thermometer prior to testing. The accuracy of the water bath was ±0.2 ℃, while the accuracy of the tags was ±0.5 ℃ within the manufacturer specified range. To calculate the absolute temperature deviation, for each tag at each test temperature, the mean tag and water bath temperature was calculated. The absolute difference between the mean tag temperature and water bath temperature was taken. For each thermal tag, the mean of the absolute temperature deviations (across all test temperatures) was then calculated. Thermal tags are ordered alphanumerically.

| **Thermal Tag ID** | **Mean absolute temperature deviation from water bath (℃)** |
| --- | --- |
| 3D9.20D4817F25 | 0.4 |
| 3D9.20D4817F30 | 0.4 |
| 3D9.20D4817F31 | 0.4 |
| 3D9.20D4A56413 | 0.5 |
| 3D9.20D4A5643E | 0.5 |
| 3D9.20D4A56459 | 0.6 |
| 3D9.20D4A5646A | 0.3 |
| 3D9.20D4A5648B | 0.5 |
| 3D9.20D4A56502 | 0.4 |
| 3D9.20D4A56520 | 0.5 |
| 3D9.20D4A565FA | 0.3 |
| 3D9.20D4A566C5 | 0.4 |
| 3D9.20D4A56738 | 0.4 |
| 3D9.20D4A81BE1 | 0.3 |
| 3D9.20D4A81F88 | 0.4 |
| 3D9.20D4A81FF7 | 0.3 |
| 3D9.20D4A82022 | 0.3 |
| 3D9.20D4A8213D | 0.4 |
| 3D9.20D4A8213F | 0.3 |
| 3D9.20D4A82287 | 0.3 |
| 3D9.20D4A82296 | 0.3 |

**Table S3.** Date, replicate number, and status of the thermal feeder during the supplemental food manipulation experiment (from 15 December 2023 to 24 February 2024). Every three days the feeder was visited to retrieve data, replace batteries, and refill or empty the feeder as needed as well as to equalize the amount of disturbance occurring at each of the feeders in the study site regardless of feeder status.

| **Date** | **Replicate** | **Feeder status** | **Feeder visit** |
| --- | --- | --- | --- |
| 15-12-2023 | 1 | Emptied  12:50:00 PM | Yes |
| 16-12-2023 | 1 | Empty | No |
| 17-12-2023 | 1 | Empty | No |
| 18-12-2023 | 1 | Empty | Yes |
| 19-12-2023 | 1 | Empty | No |
| 20-12-2023 | 1 | Empty | No |
| 21-12-2023 | 1 | Refilled  12:31:00 PM | Yes |
| 22-12-2023 | 1 | Full | No |
| 23-12-2023 | 1 | Full | No |
| 24-12-2023 | 1 | Full | Yes |
| 25-12-2023 | 1 | Full | No |
| 26-12-2023 | 1 | Full | No |
| 27-12-2023 | 2 | Emptied  10:57:00 AM | Yes |
| 28-12-2023 | 2 | Empty | No |
| 29-12-2023 | 2 | Empty | No |
| 30-12-2023 | 2 | Empty | Yes |
| 31-12-2023 | 2 | Empty | No |
| 01-01-2024 | 2 | Empty | No |
| 02-01-2024 | 2 | Refilled  10:21:00 AM | Yes |
| 03-01-2024 | 2 | Full | No |
| 04-01-2024 | 2 | Full | No |
| 05-01-2024 | 2 | Full | Yes |
| 06-01-2024 | 2 | Full | No |
| 07-01-2024 | 2 | Full | No |
| 08-01-2024 | 3 | Emptied  12:49:00 PM | Yes |
| 09-01-2024 | 3 | Empty | No |
| 10-01-2024 | 3 | Empty | No |
| 11-01-2024 | 3 | Empty | Yes |
| 12-01-2024 | 3 | Empty | No |
| 13-01-2024 | 3 | Empty | No |
| 14-01-2024 | 3 | Refilled  12:44:00 PM | Yes |
| 15-01-2024 | 3 | Full | No |
| 16-01-2024 | 3 | Full | No |
| 17-01-2024 | 3 | Full | Yes |
| 18-01-2024 | 3 | Full | No |
| 19-01-2024 | 3 | Full | No |
| 20-01-2024 | 4 | Emptied  11:00:00 AM | Yes |
| 21-01-2024 | 4 | Empty | No |
| 22-01-2024 | 4 | Empty | No |
| 23-01-2024 | 4 | Empty | Yes |
| 24-01-2024 | 4 | Empty | No |
| 25-01-2024 | 4 | Empty | No |
| 26-01-2024 | 4 | Refilled  13:04:00 PM | Yes |
| 27-01-2024 | 4 | Full | No |
| 28-01-2024 | 4 | Full | No |
| 29-01-2024 | 4 | Full | Yes |
| 30-01-2024 | 4 | Full | No |
| 31-01-2024 | 4 | Full | No |
| 01-02-2024 | 5 | Emptied  10:26:00 AM | Yes |
| 02-02-2024 | 5 | Empty | No |
| 03-02-2024 | 5 | Empty | No |
| 04-02-2024 | 5 | Empty | Yes |
| 05-02-2024 | 5 | Empty | No |
| 06-02-2024 | 5 | Empty | No |
| 07-02-2024 | 5 | Refilled  10:51:00 AM | Yes |
| 08-02-2024 | 5 | Full | No |
| 09-02-2024 | 5 | Full | No |
| 10-02-2024 | 5 | Full | Yes |
| 11-02-2024 | 5 | Full | No |
| 12-02-2024 | 5 | Full | No |
| 13-02-2024 | 6 | Emptied  10:52:00 AM | Yes |
| 14-02-2024 | 6 | Empty | No |
| 15-02-2024 | 6 | Empty | No |
| 16-02-2024 | 6 | Empty | Yes |
| 17-02-2024 | 6 | Empty | No |
| 18-02-2024 | 6 | Empty | No |
| 19-02-2024 | 6 | Refilled  10:57:00 AM | Yes |
| 20-02-2024 | 6 | Full | No |
| 21-02-2024 | 6 | Full | No |
| 22-02-2024 | 6 | Full | Yes |
| 23-02-2024 | 6 | Full | No |
| 24-02-2024 | 6 | Full | No |

**Table S4.** Effects of supplemental food availability, hourly ambient T_a_, daylength and time of day on visit T_sub_ in active chickadees in winter. Models were run on datasets that applied different data filtering to ensure that subjective data filtering decisions did not unduly influence model inferences. Model effects presented are the mode of the posterior distribution with 95% CrI, while the adjusted repeatability estimates are the point estimates and 95% CrI. The proportion of estimates overlapping zero (pr) are provided where applicable.

|  | **Outlier individuals retained, spurious datapoints removed** | **Outlier individuals retained,  spurious datapoints retained** | **Outlier individuals removed,  spurious datapoints retained** |
| --- | --- | --- | --- |
| **Fixed effect** | **β (95% CrI)** | | |
| Intercept^a^ | 41.10 (40.29, 42.50) | 41.46 (40.19, 42.39) | 41.97 (41.60, 42.44) |
| Feeder.status: Empty | -0.74 (-0.95, -0.52) | -0.69 (-0.94, -0.52) | -0.37 (-0.55, -0.20) |
| Days since transition^b^ | -0.05 (-0.06, -0.02) | -0.04 (-0.06, -0.02) | -0.02 (-0.05, -0.01) |
| Days since transition × Feeder.status: Empty | -0.02 (-0.13, 0.04)  pr = 0.20 | -0.03 (-0.13, 0.05)  pr = 0.23 | -0.03 (-0.10, 0.05)  pr = 0.24 |
| Hourly T_a_^c^ | 0.27 (0.24, 0.28) | 0.26 (0.24, 0.29) | 0.23 (0.21, 0.24) |
| Hourly T_a_^c^ × Feeder.status: Empty | 0.40 (0.29, 0.51) | 0.41 (0.29, 0.51) | 0.20 (0.09, 0.27) |
| Daylength (hr)^d^ | 0.27 (0.04, 0.59) | 0.35 (0.02, 0.57) | 0.42 (0.14, 0.56) |
| Daylength (hr) ^d^ × Feeder.status: Empty | -0.18 (-0.28, -0.07) | -0.17 (-0.28, -0.07) | -0.12 (-0.20, -0.05) |
| Time of day^e^ | 0.26 (0.23, 0.27) | 0.25 (0.23, 0.27) | 0.22 (0.21, 0.23) |
| Time of day^e^ × Feeder.status: Empty | -0.15 (-0.26, -0.02) | -0.17 (-0.29, -0.02) | 0.02 (-0.11, 0.09) |
| Time of day^2e^ | -0.25 (-0.29, -0.21) | -0.25 (-0.29, -0.21) | -0.23 (-0.25, -0.18) |
| Time of day^2e^ × Feeder.status: Empty | 0.76 (0.44, 1.06) | 0.69 (0.37, 1.05) | 0.56 (0.27, 0.74) |
| **Random effect** | **σ (95% CrI)** | | |
| Individual ID | 4.97 (4.94, 5.01) | 4.97 (4.93, 5.01) | 0.47 (0.46, 0.47) |
| Replicate | 0.11 (0.05, 0.23) | 0.11 (0.05, 0.22) | 0.07 (0.04, 0.17) |
| Residual | 0.28 (0.28, 0.29) | 0.30 (0.29, 0.30) | 0.16 (0.15, 0.16) |
| **Repeatability^f^** | ***Adjusted r* (95% CI)** | | |
| Individual ID | 0.92 (0.84, 0.96) | 0.92 (0.81, 0.96) | 0.65 (0.46, 0.79) |

^a^ Reference level for Feeder.status was set to “Full” such that intercepts were estimated at when the feeder was full.

^b^ Day since transition was left-zeroed and standardized such that intercepts were estimated for the first full day the supplemental feeder was full or empty and estimates represent the effect of 2 SD (3.16 days).

^c^ Hourly T_a_ was standardized and left-zeroed such that intercepts were estimated at the coldest T_a_ (-34.0 ℃) and estimated effects represent as change in 2 SD (16.49 ℃)

^d^ Daylength was centered and standardized such the intercepts were estimated at the mean daylength 8.56 hr and estimated effects represent a changed 2 SD (1.96 hr of daylength)

^e^ Time of day (hr) was centred and standardized such that intercepts were estimated at noon (12:00 pm) and estimated effects represent a change in 2 SD (6.63 hr)

^f^ Adjusted repeatability was calculated using the rptR package (Stoffel et al., 2017).


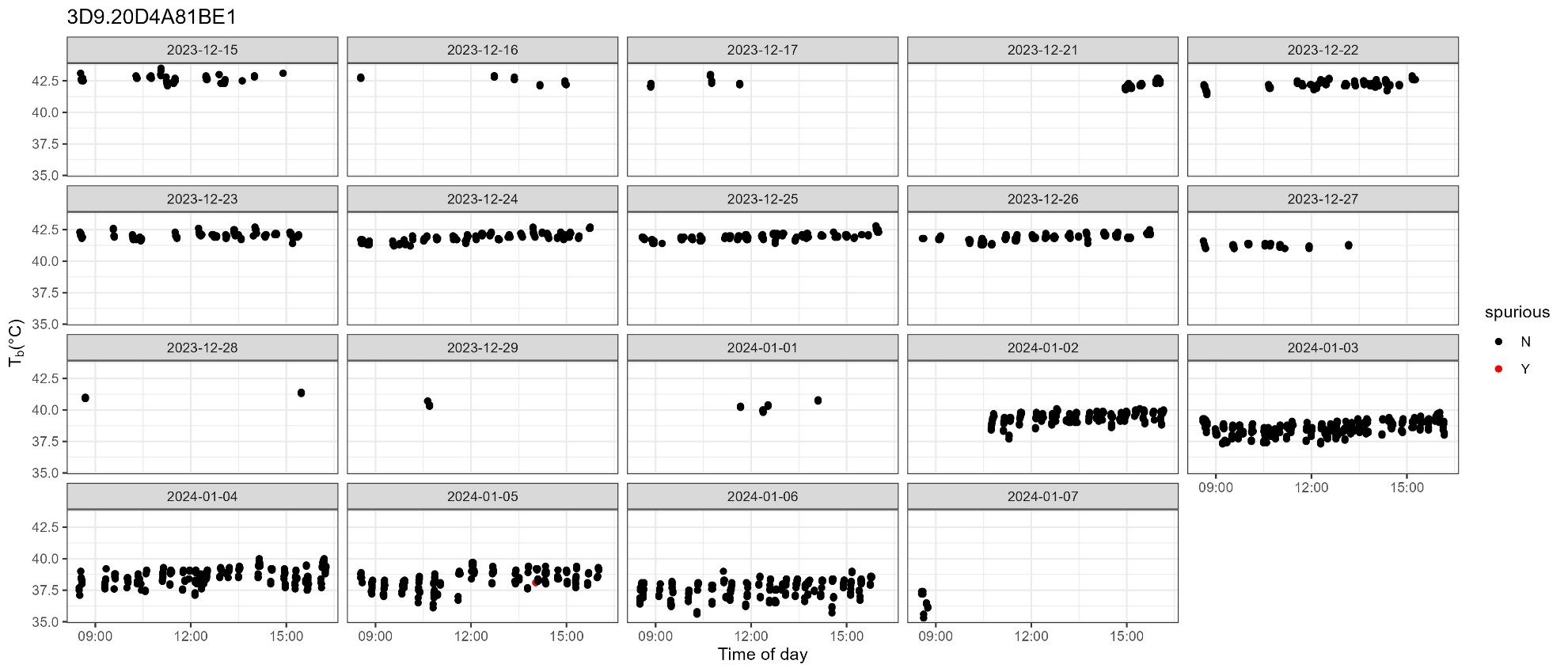


# **Figure S1.** Daily plots of subcutaneous body temperatures (T _sub_) in relation to time of day for individual 3D9.20D4A81BE1 while they were detected at the thermal feeder during the food manipulation experiment. Black dots represent T _sub_ detections that were not identified as spurious while red dots represent T_sub_ detections that were identified as spurious.


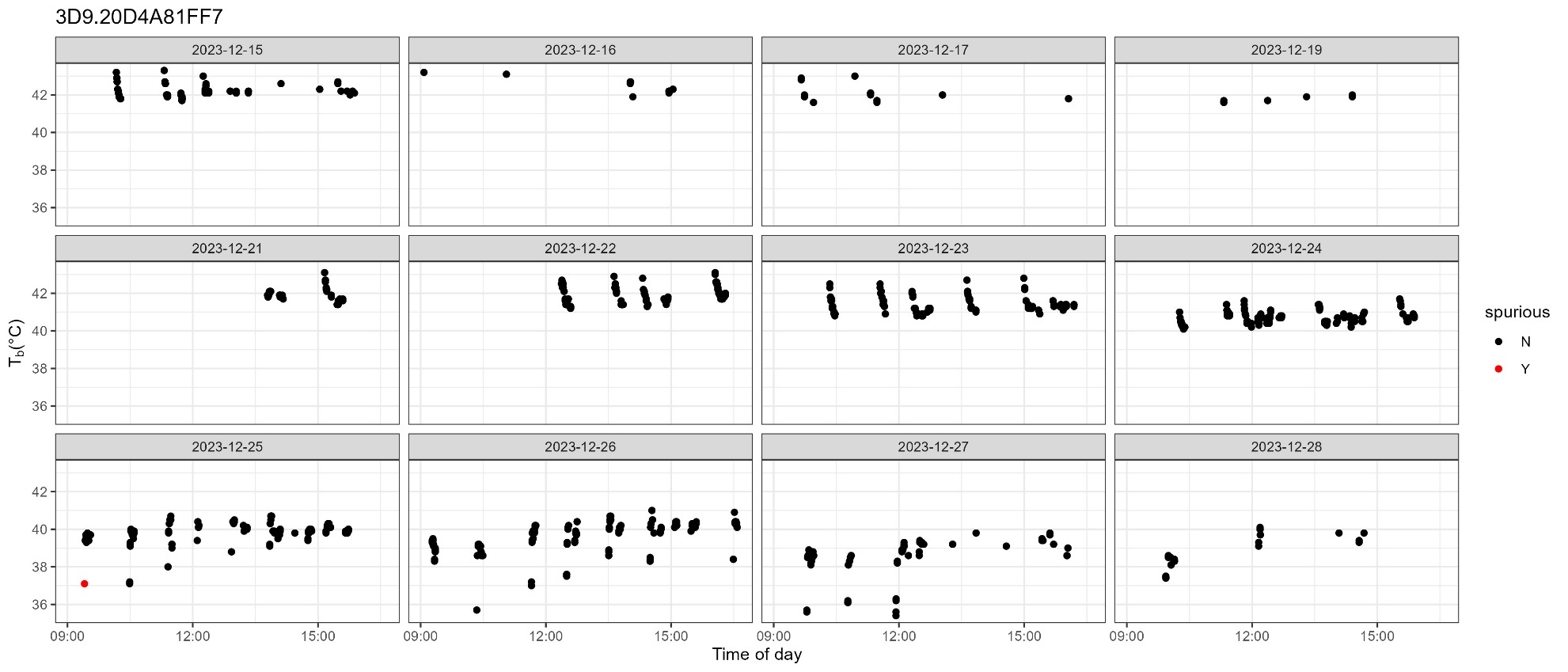


**Figure S2.** Daily plots of subcutaneous body temperatures (T _sub_) in relation to time of day for individual 3D9.20D4A81FF7 while they were detected at the thermal feeder during the food manipulation experiment. Black dots represent T _sub_ detections that were not identified as spurious while red dots represent T_sub_ detections that were identified as spurious.


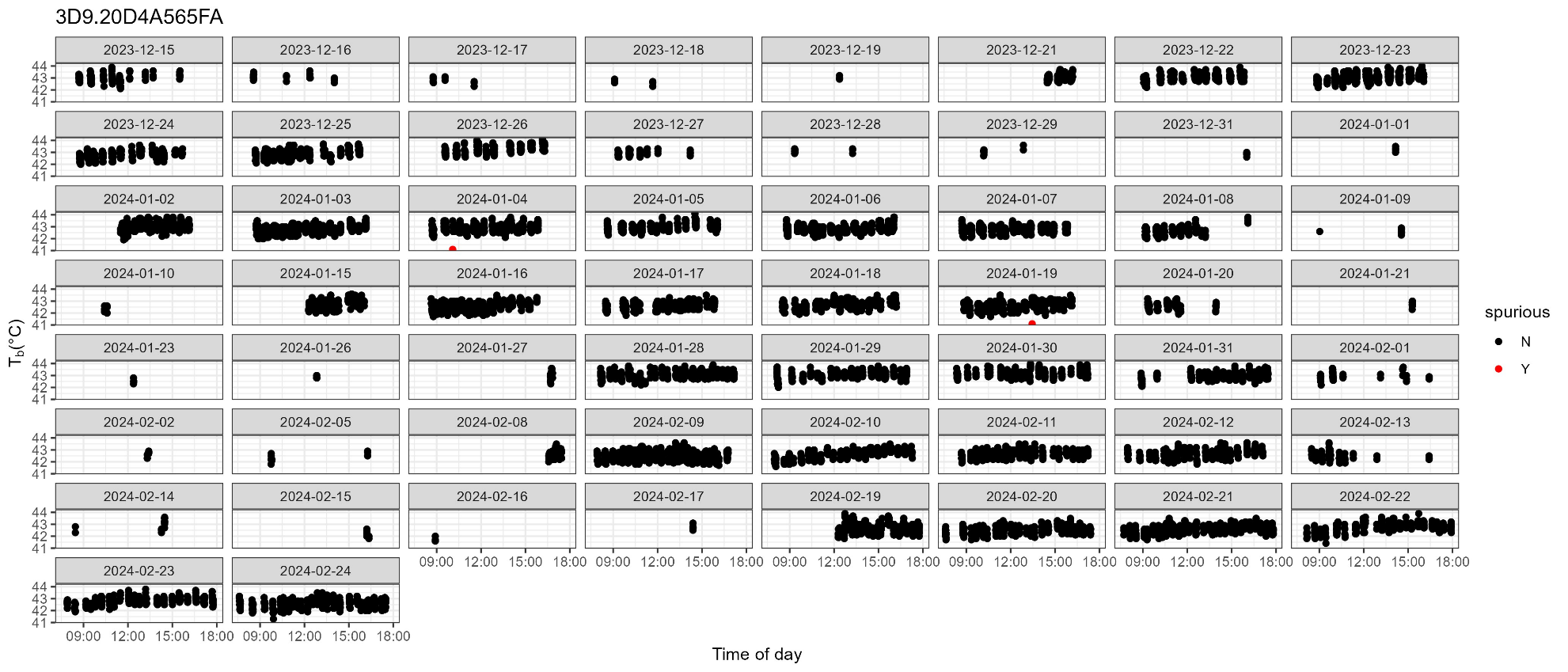


**Figure S3.** Daily plots of subcutaneous body temperatures (T _sub_) in relation to time of day for individual 3D9.20D4A565FA while they were detected at the thermal feeder during the food manipulation experiment. Black dots represent T_sub_ detections that were not identified as spurious while red dots represent T_sub_ detections that were identified as spurious.


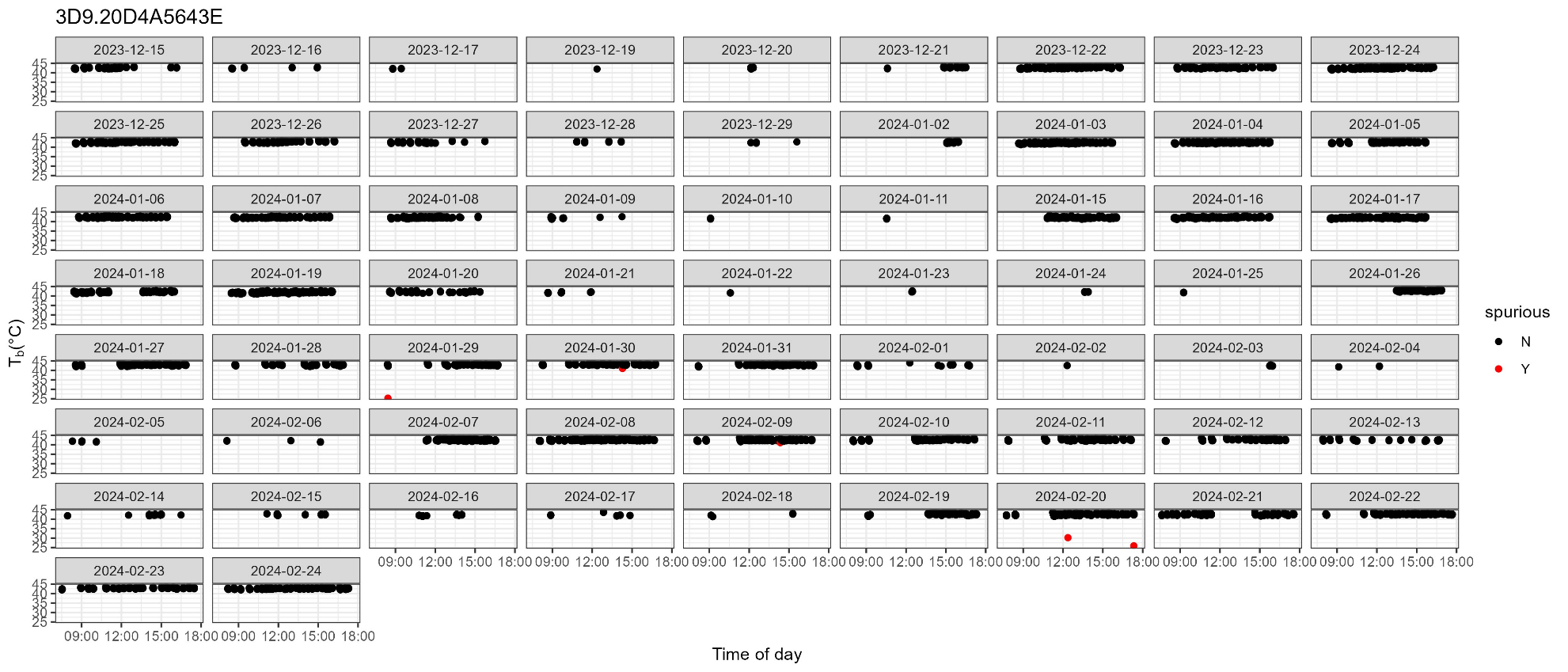


**Figure S4.** Daily plots of subcutaneous body temperatures (T _sub_) in relation to time of day for individual 3D9.20D4A5643E while they were detected at the thermal feeder during the food manipulation experiment. Black dots represent T_sub_ detections that were not identified as spurious while red dots represent T_sub_ detections that were identified as spurious.


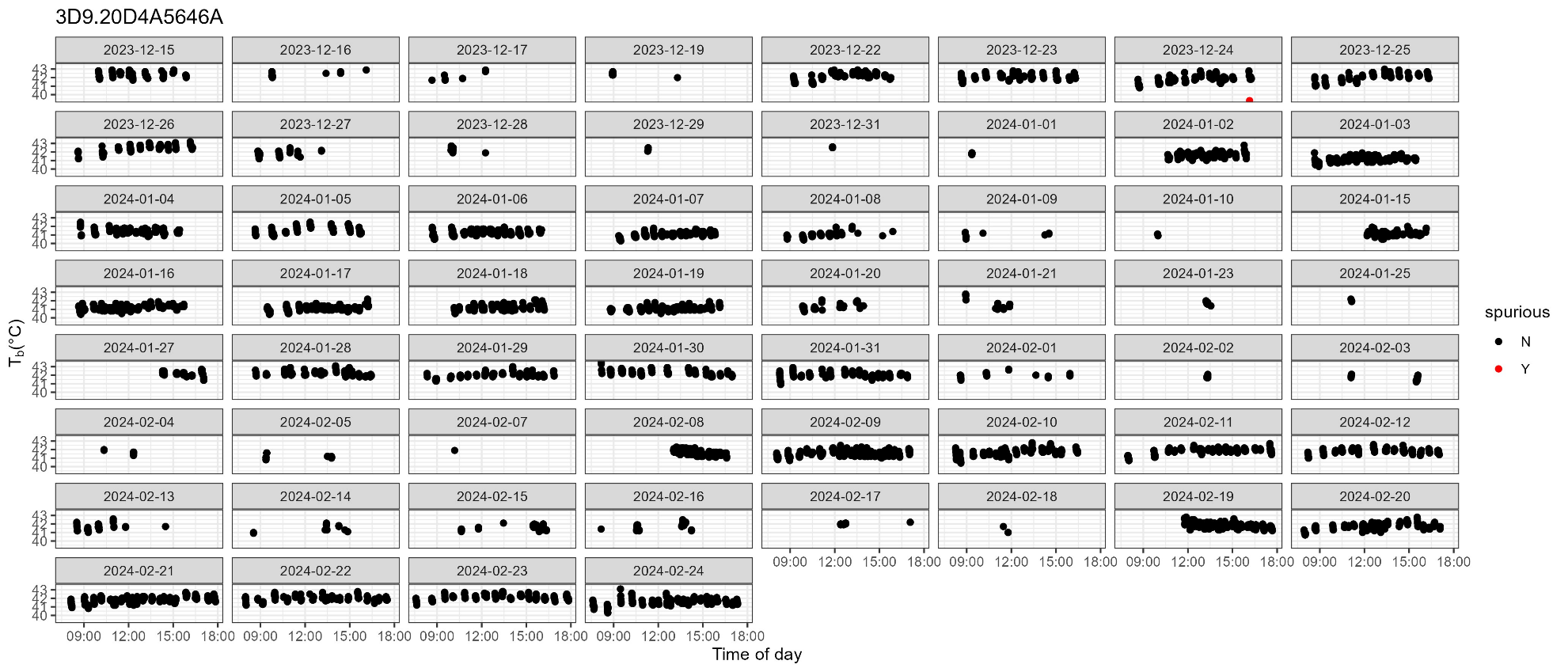


**Figure S5.** Daily plots of subcutaneous body temperatures (T _sub_) in relation to time of day for individual 3D9.20D4A5646A while they were detected at the thermal feeder during the food manipulation experiment. Black dots represent T_sub_ detections that were not identified as spurious while red dots represent T_sub_ detections that were identified as spurious.


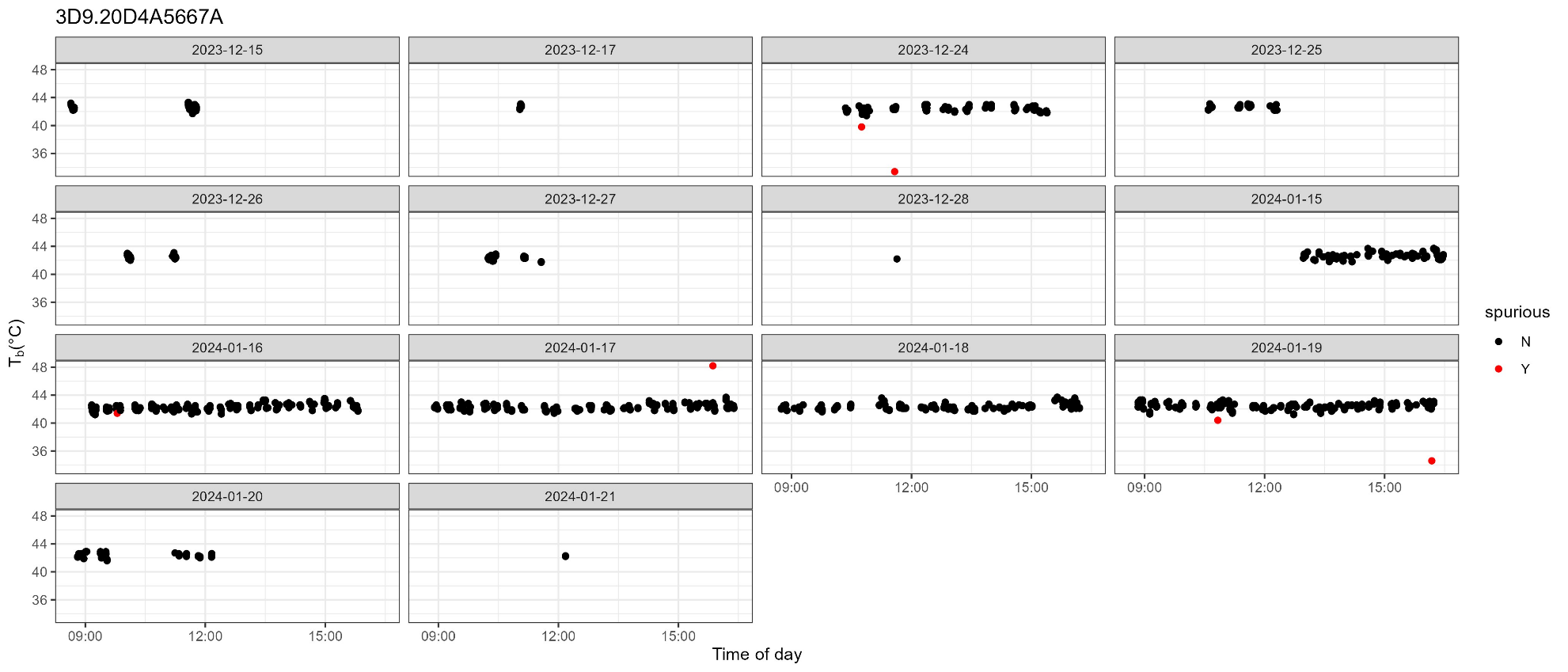


**Figure S6.** Daily plots of subcutaneous body temperatures (T _sub_) in relation to time of day for individual 3D9.20D45667A while they were detected at the thermal feeder during the food manipulation experiment. Black dots represent T_sub_ detections that were not identified as spurious while red dots represent T_sub_ detections that were identified as spurious.


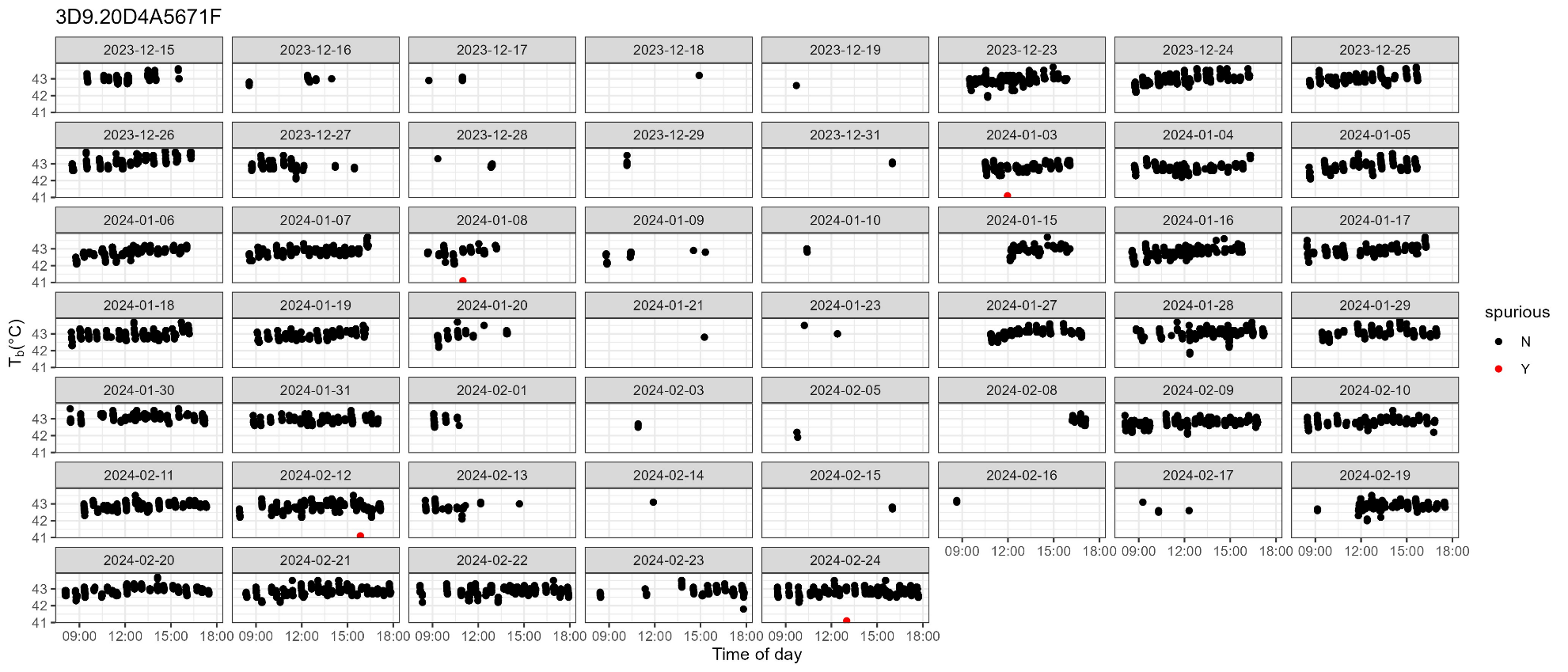


**Figure S7.** Daily plots of subcutaneous body temperatures (T _sub_) in relation to time of day for individual 3D9.20D4A5671F while they were detected at the thermal feeder during the food manipulation experiment. Black dots represent T_sub_ detections that were not identified as spurious while red dots represent T_sub_ detections that were identified as spurious.


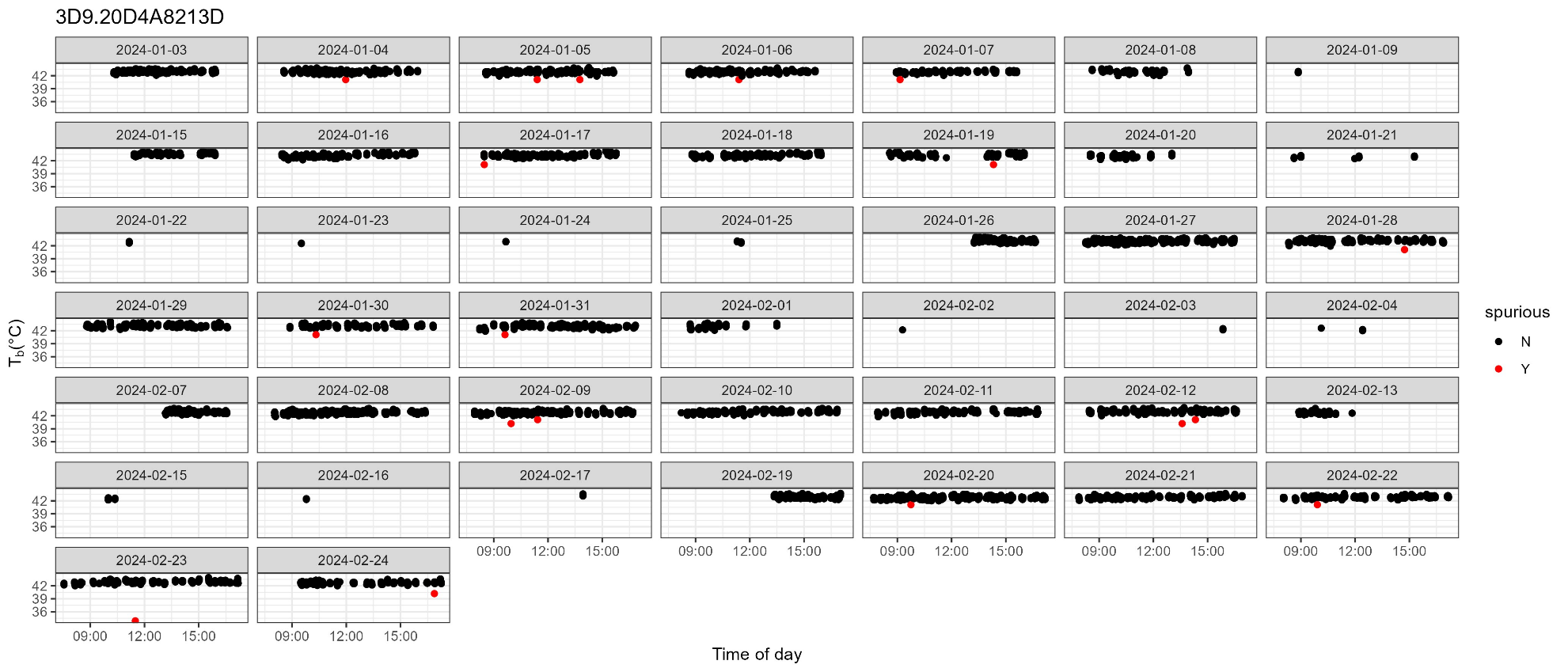


**Figure S8.** Daily plots of subcutaneous body temperatures (T _sub_) in relation to time of day for individual 3D9.20D4A8213D while they were detected at the thermal feeder during the food manipulation experiment. Black dots represent T_sub_ detections that were not identified as spurious while red dots represent T_sub_ detections that were identified as spurious.


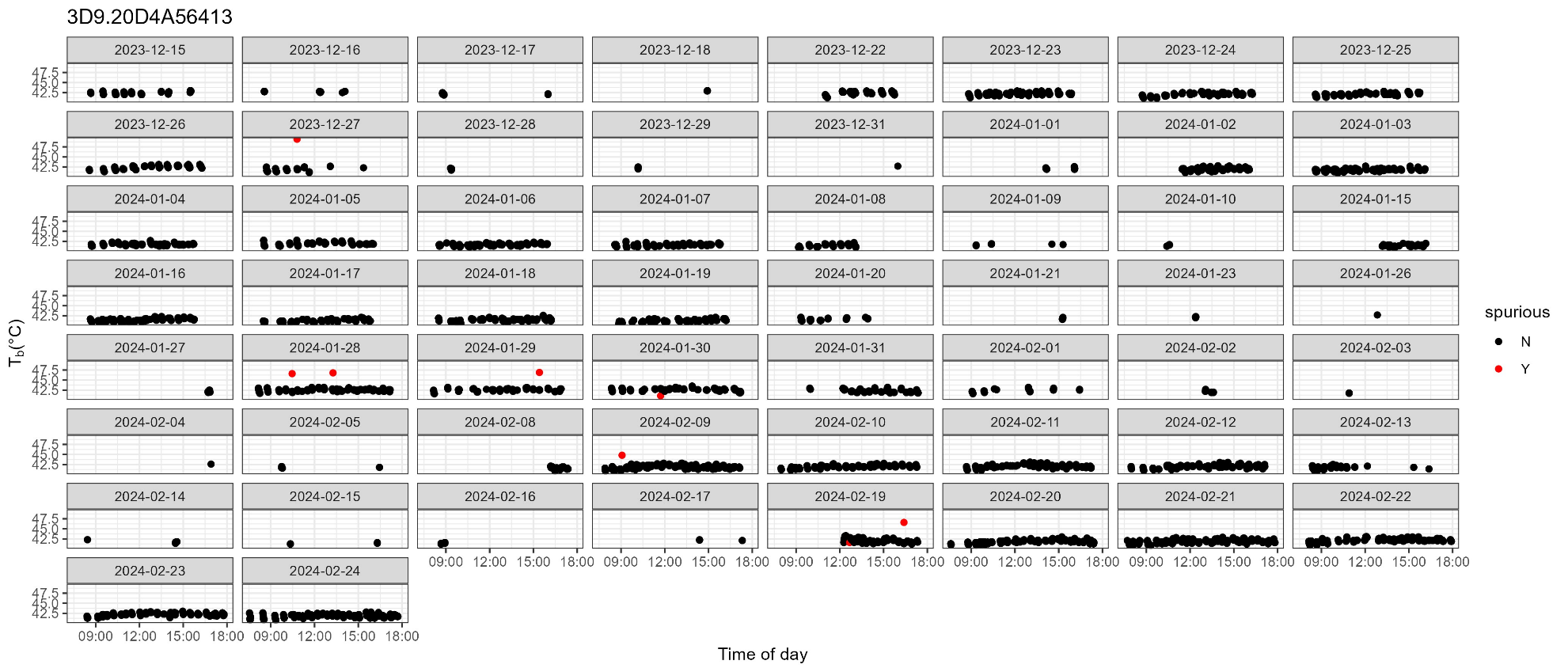


**Figure S9.** Daily plots of subcutaneous body temperatures (T _sub_) in relation to time of day for individual 3D9.20D4A56413 while they were detected at the thermal feeder during the food manipulation experiment. Black dots represent T_sub_ detections that were not identified as spurious while red dots represent T_sub_ detections that were identified as spurious.


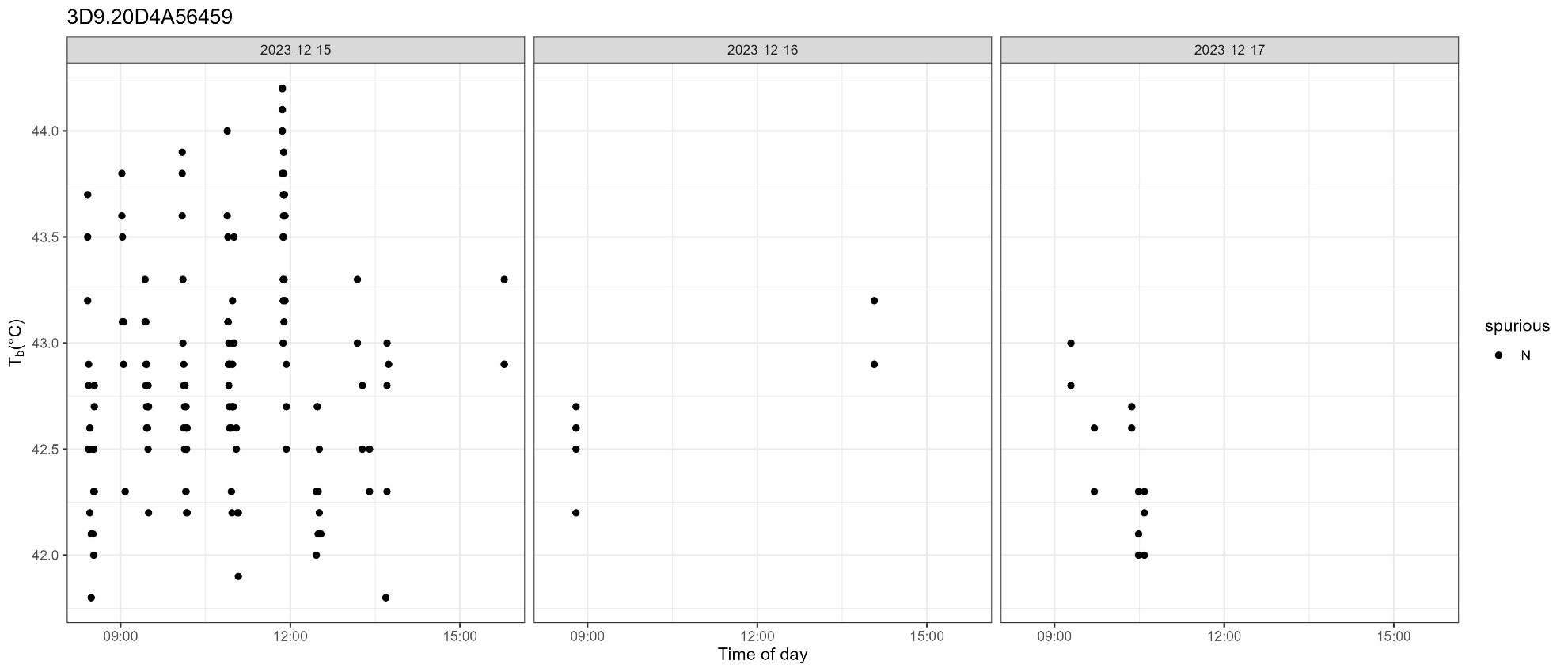


**Figure S10.** Daily plots of subcutaneous body temperatures (T _sub_) in relation to time of day for individual 3D9.20D4A56459 while they were detected at the thermal feeder during the food manipulation experiment. Black dots represent T_sub_ detections that were not identified as spurious while red dots represent T_sub_ detections that were identified as spurious. Note: no detections were identified as spurious.


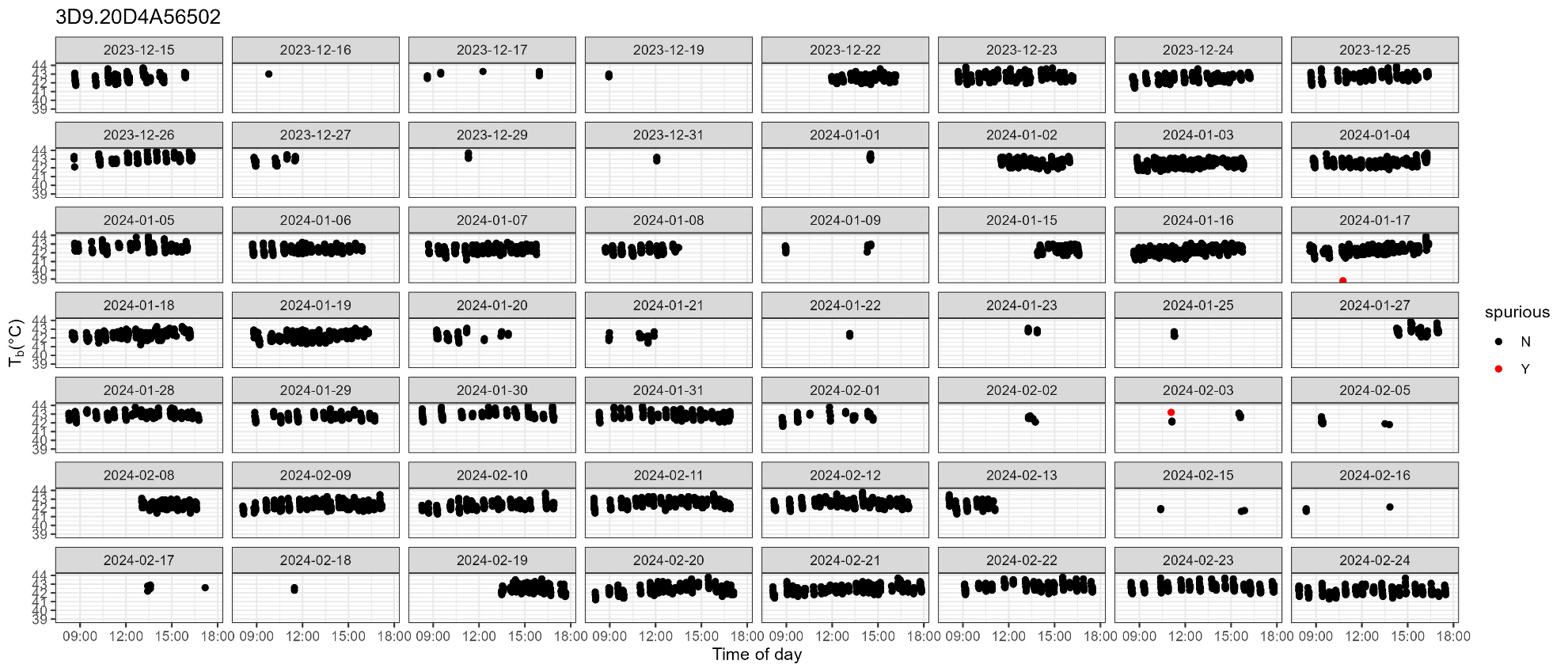


**Figure S11.** Daily plots of subcutaneous body temperatures (T _sub_) in relation to time of day for individual 3D9.20D4A56502 while they were detected at the thermal feeder during the food manipulation experiment. Black dots represent T_sub_ detections that were not identified as spurious while red dots represent T_sub_ detections that were identified as spurious.


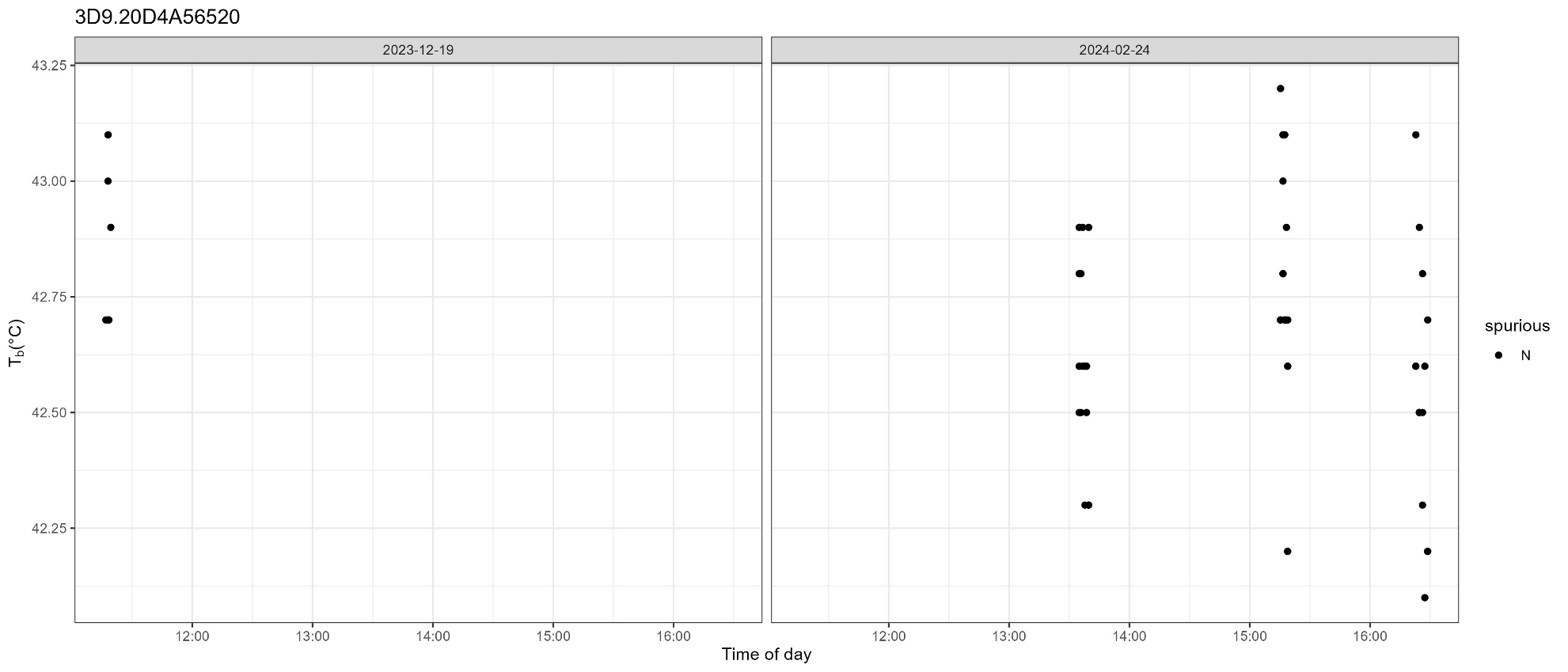


**Figure S12.** Daily plots of subcutaneous body temperatures (T _sub_) in relation to time of day for individual 3D9.20D4A56520 while they were detected at the thermal feeder during the food manipulation experiment. Black dots represent T_sub_ detections that were not identified as spurious while red dots represent T_sub_ detections that were identified as spurious. Note: no detections were identified as spurious.


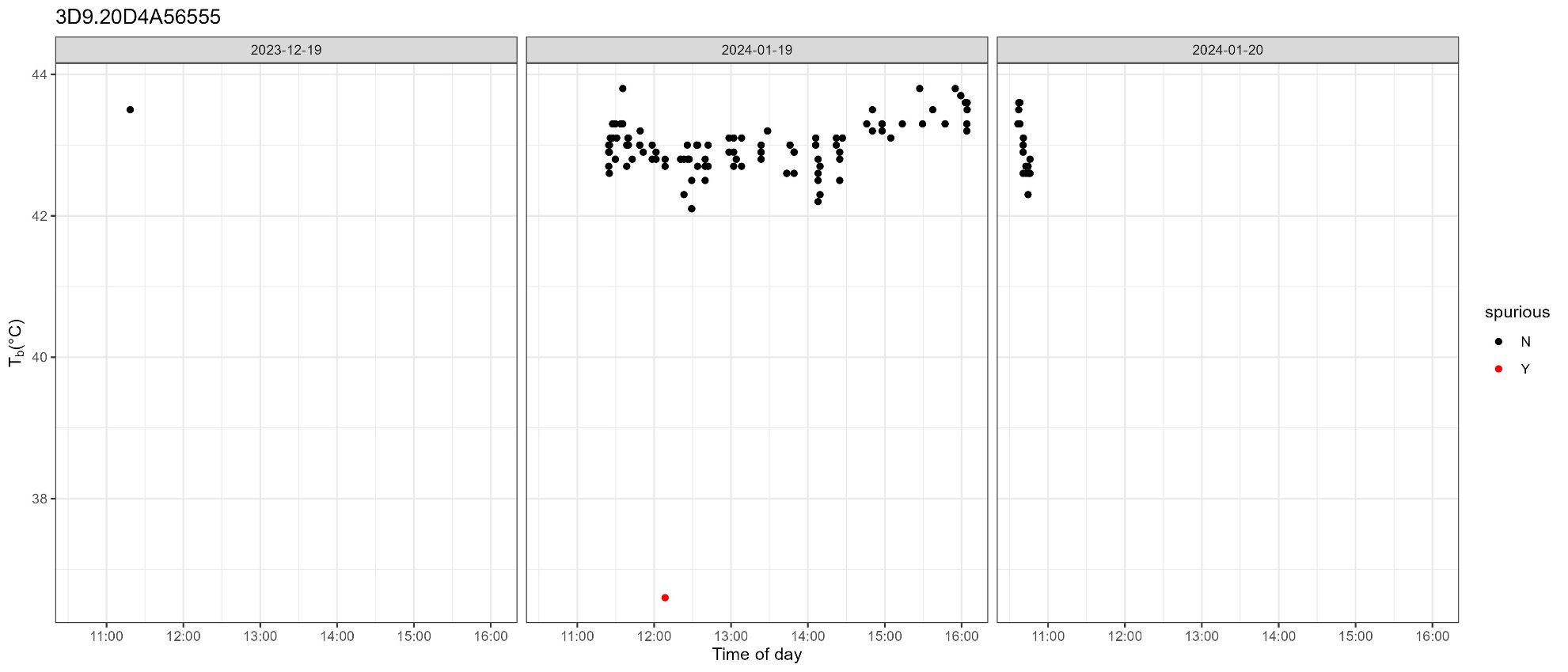


**Figure S13.** Daily plots of subcutaneous body temperatures (T _sub_) in relation to time of day for individual 3D9.20D4A56555 while they were detected at the thermal feeder during the food manipulation experiment. Black dots represent T_sub_ detections that were not identified as spurious while red dots represent T_sub_ detections that were identified as spurious.


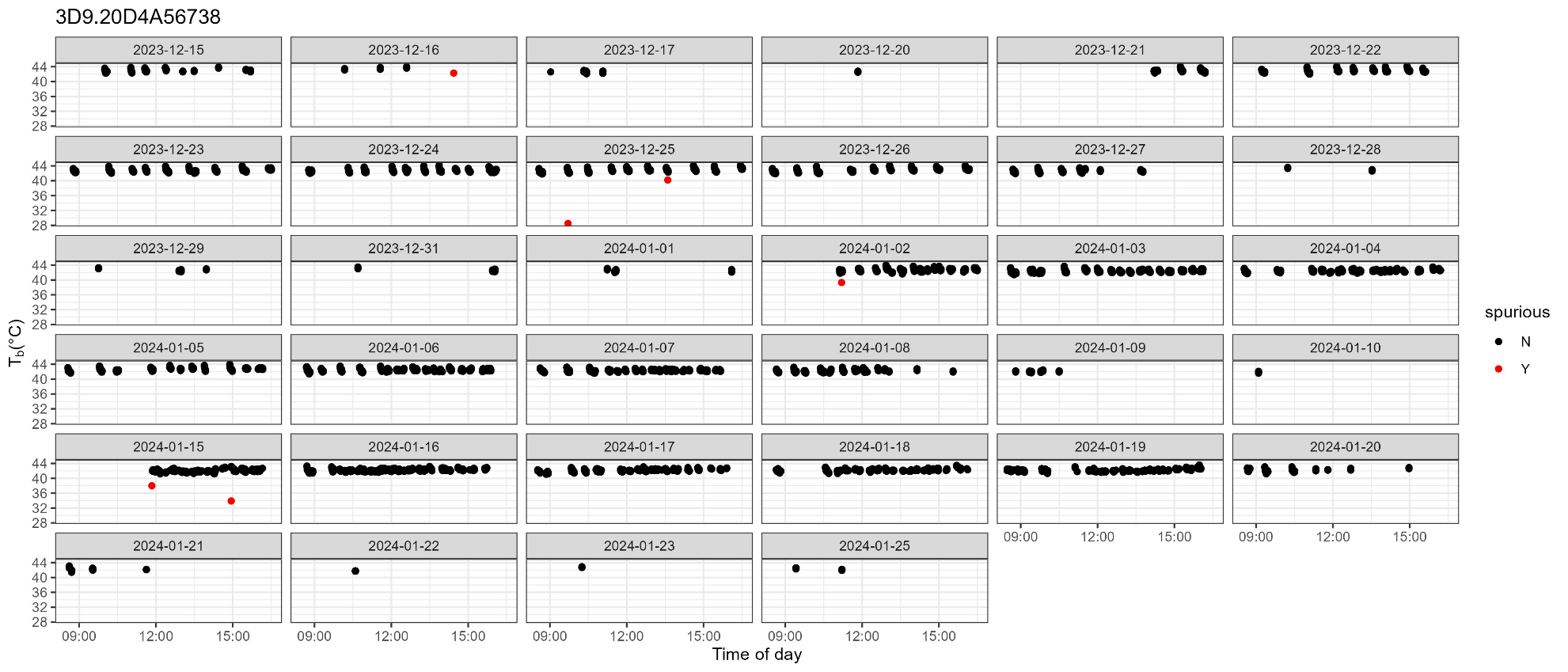


**Figure S14.** Daily plots of subcutaneous body temperatures (T _sub_) in relation to time of day for individual 3D9.20D4A56738 while they were detected at the thermal feeder during the food manipulation experiment. Black dots represent T_sub_ detections that were not identified as spurious while red dots represent T_sub_ detections that were identified as spurious.


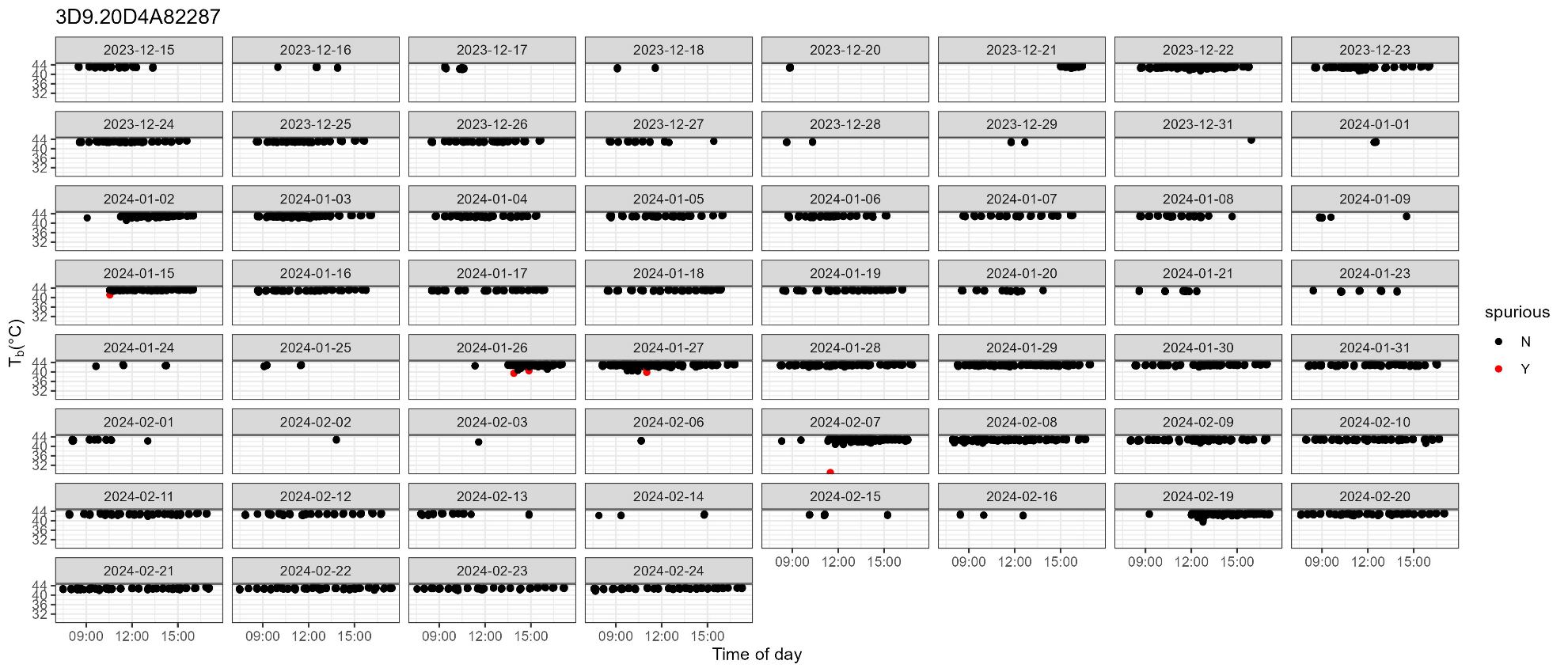


**Figure S15.** Daily plots of subcutaneous body temperatures (T _sub_) in relation to time of day for individual 3D9.20D4A82287 while they were detected at the thermal feeder during the food manipulation experiment. Black dots represent T_sub_ detections that were not identified as spurious while red dots represent T_sub_ detections that were identified as spurious.


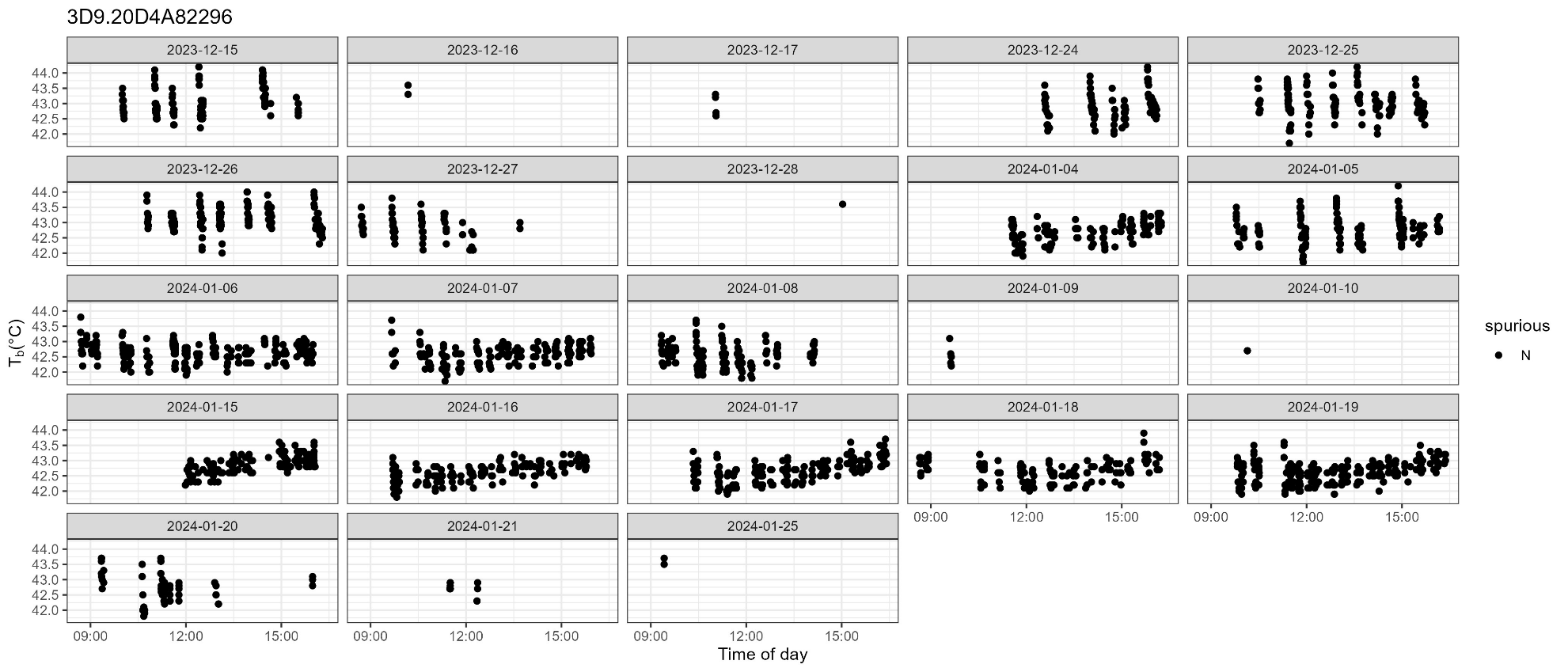


**Figure S16.** Daily plots of subcutaneous body temperatures (T _sub_) in relation to time of day for individual 3D9.20D4A82296 while they were detected at the thermal feeder the food manipulation experiment. Black dots represent T_sub_ detections that were not identified as spurious while red dots represent T_sub_ detections that were identified as spurious. Note: no detections were identified as spurious.


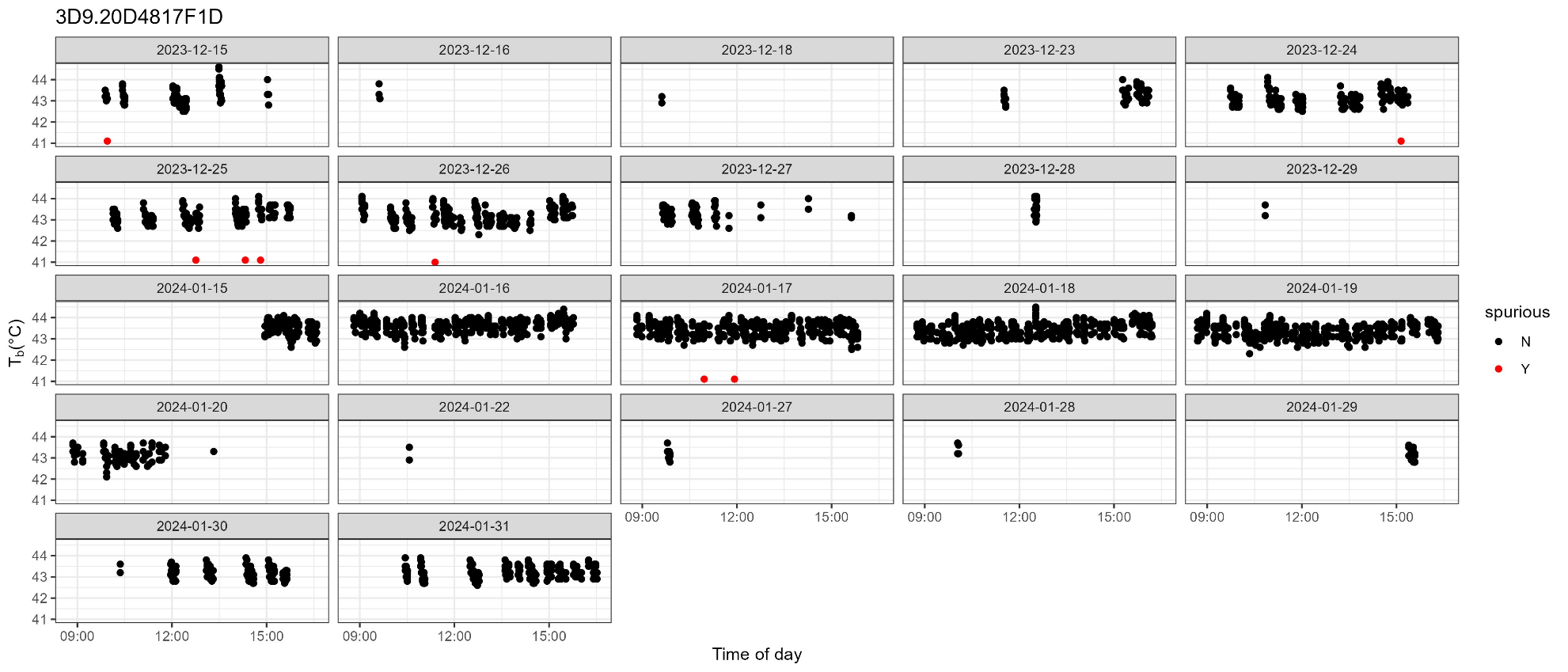


**Figure S17.** Daily plots of subcutaneous body temperatures (T _sub_) in relation to time of day for individual 3D9.20D4817F1D while they were detected at the thermal feeder during the food manipulation experiment. Black dots represent T_sub_ detections that were not identified as spurious while red dots represent T_sub_ detections that were identified as spurious.


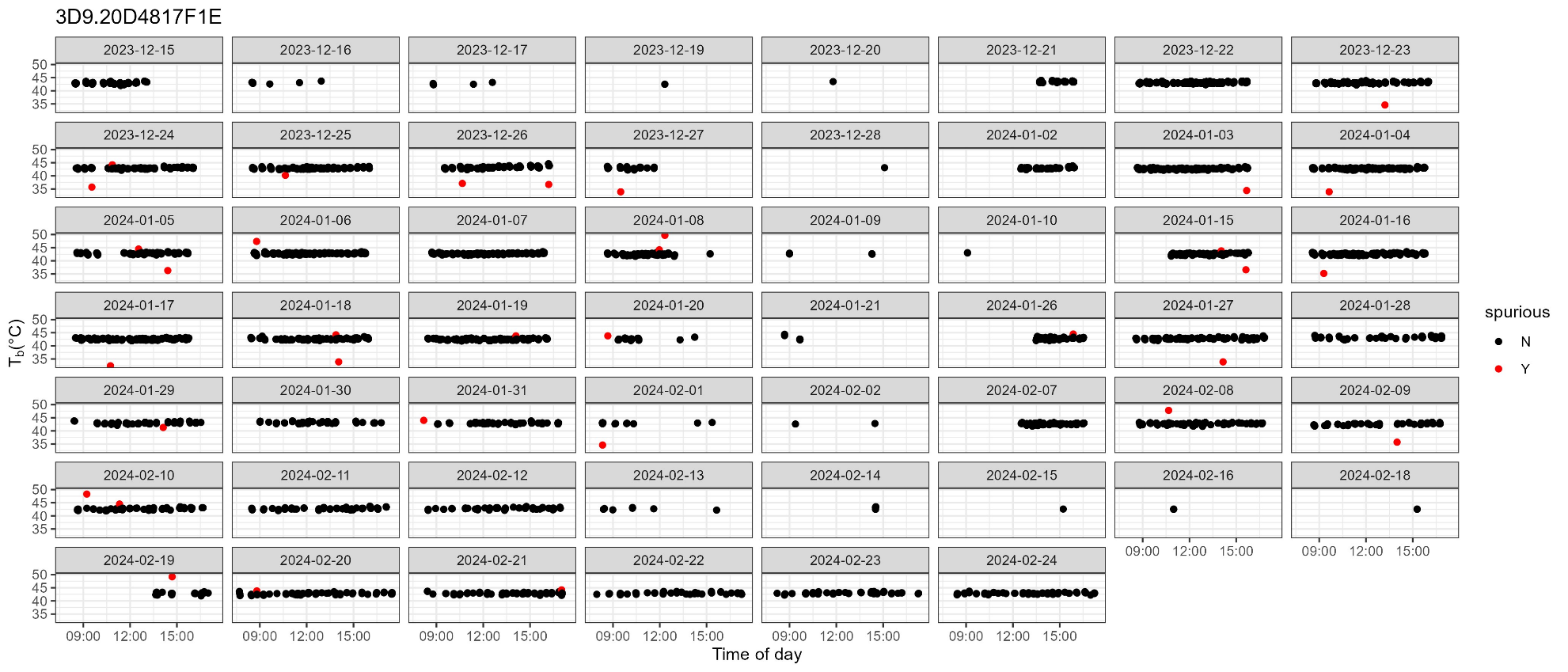


**Figure S18.** Daily plots of subcutaneous body temperatures (T _sub_) in relation to time of day for individual 3D9.20D4817F1E while they were detected at the thermal feeder during the food manipulation experiment. Black dots represent T_sub_ detections that were not identified as spurious while red dots represent T_sub_ detections that were identified as spurious.


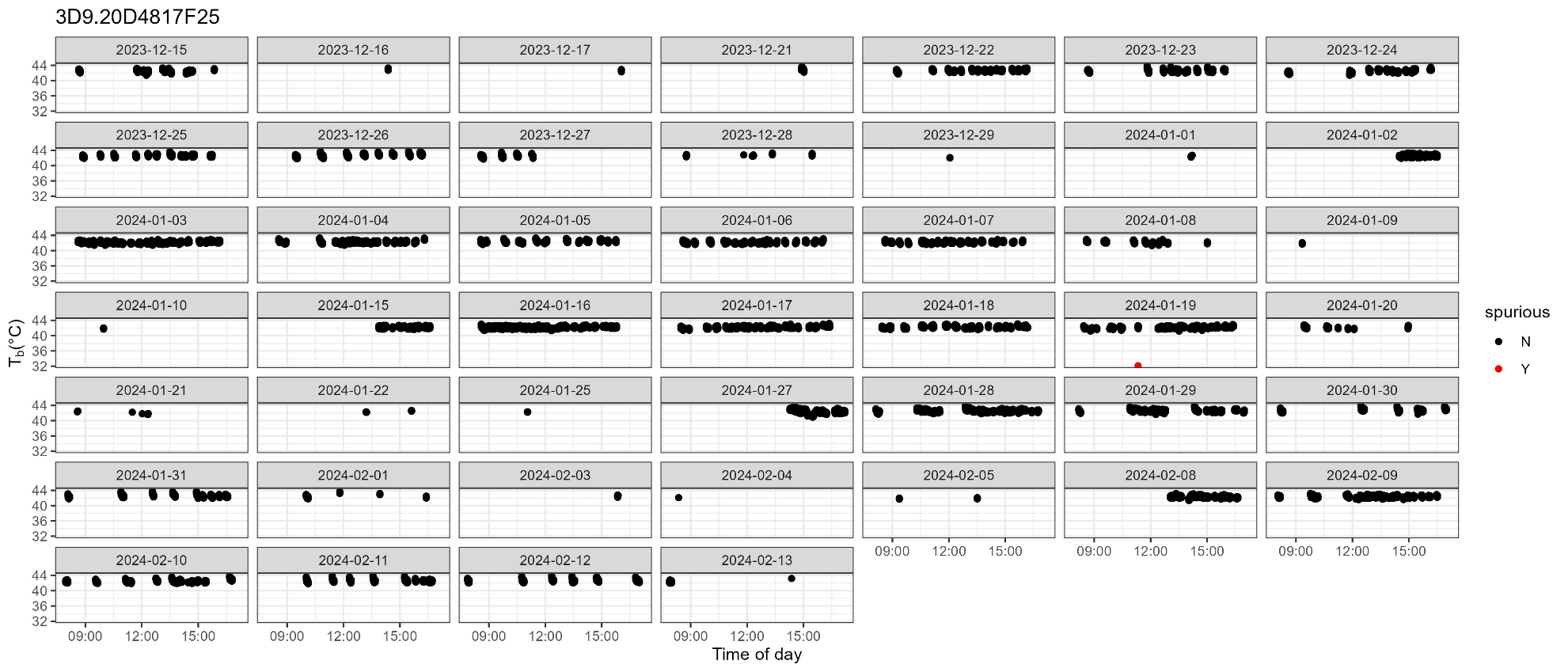


**Figure S19.** Daily plots of subcutaneous body temperatures (T _sub_) in relation to time of day for individual 3D9.20D4817F25 while they were detected at the thermal feeder during the food manipulation experiment. Black dots represent T_sub_ detections that were not identified as spurious while red dots represent T_sub_ detections that were identified as spurious.


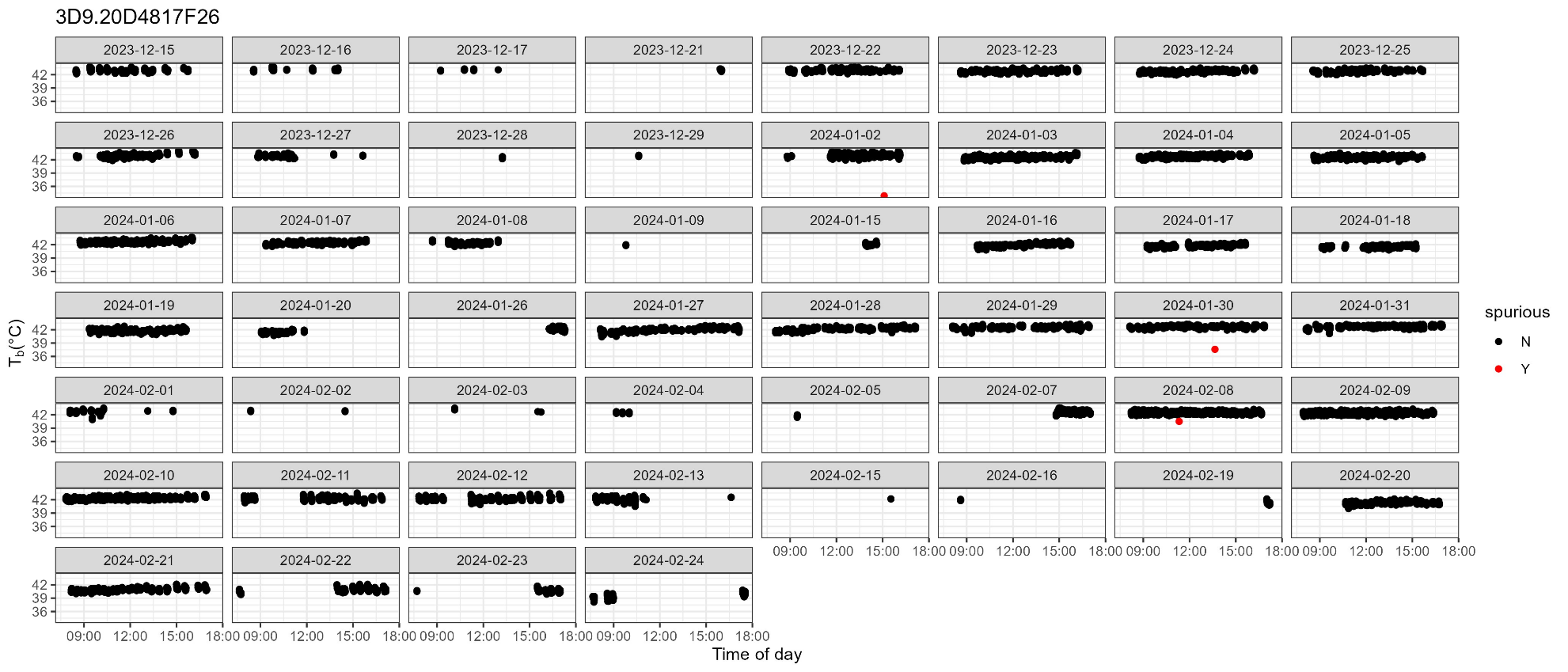


**Figure S20.** Daily plots of subcutaneous body temperatures (T _sub_) in relation to time of day for individual 3D9.20D4817F26 while they were detected at the thermal feeder during the food manipulation experiment. Black dots represent T_sub_ detections that were not identified as spurious while red dots represent T_sub_ detections that were identified as spurious.


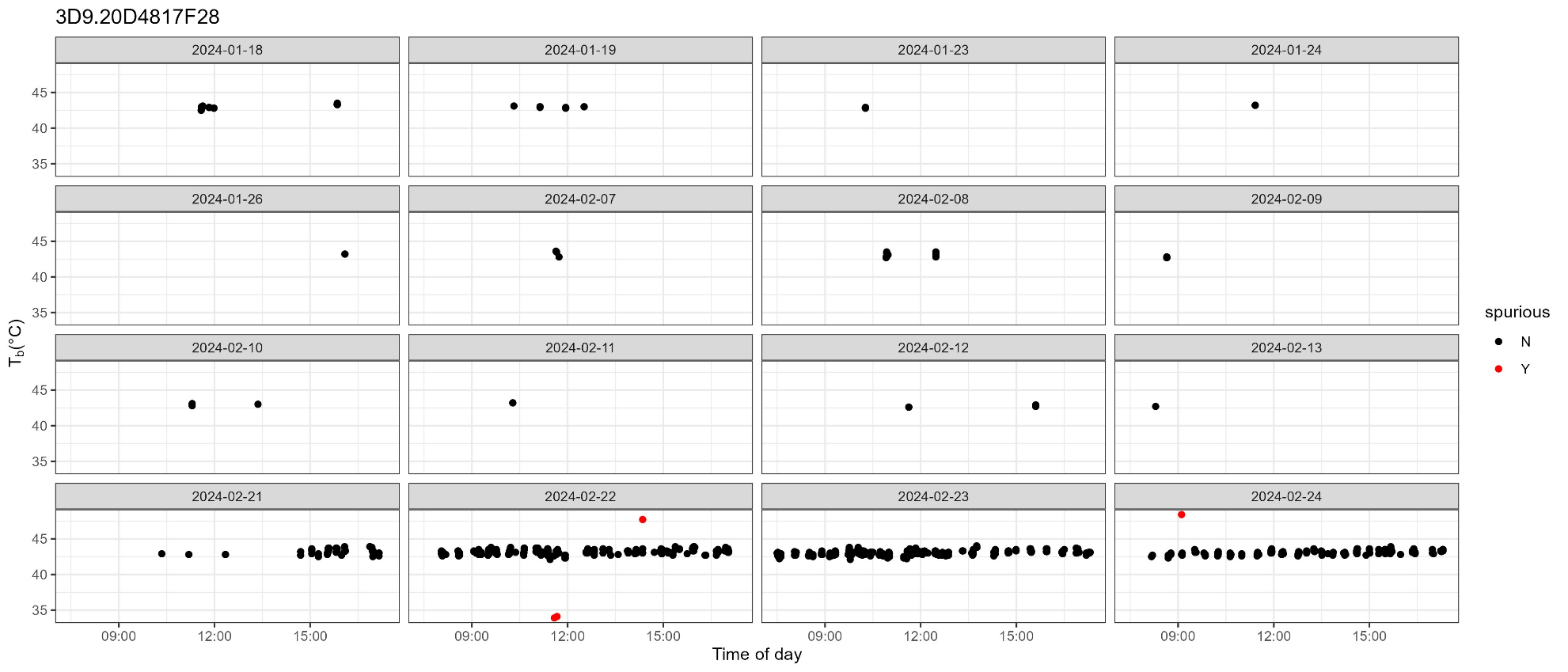


**Figure S21.** Daily plots of subcutaneous body temperatures (T _sub_) in relation to time of day for individual 3D9.20D4817F28 while they were detected at the thermal feeder during the food manipulation experiment. Black dots represent T_sub_ detections that were not identified as spurious while red dots represent T_sub_ detections that were identified as spurious.


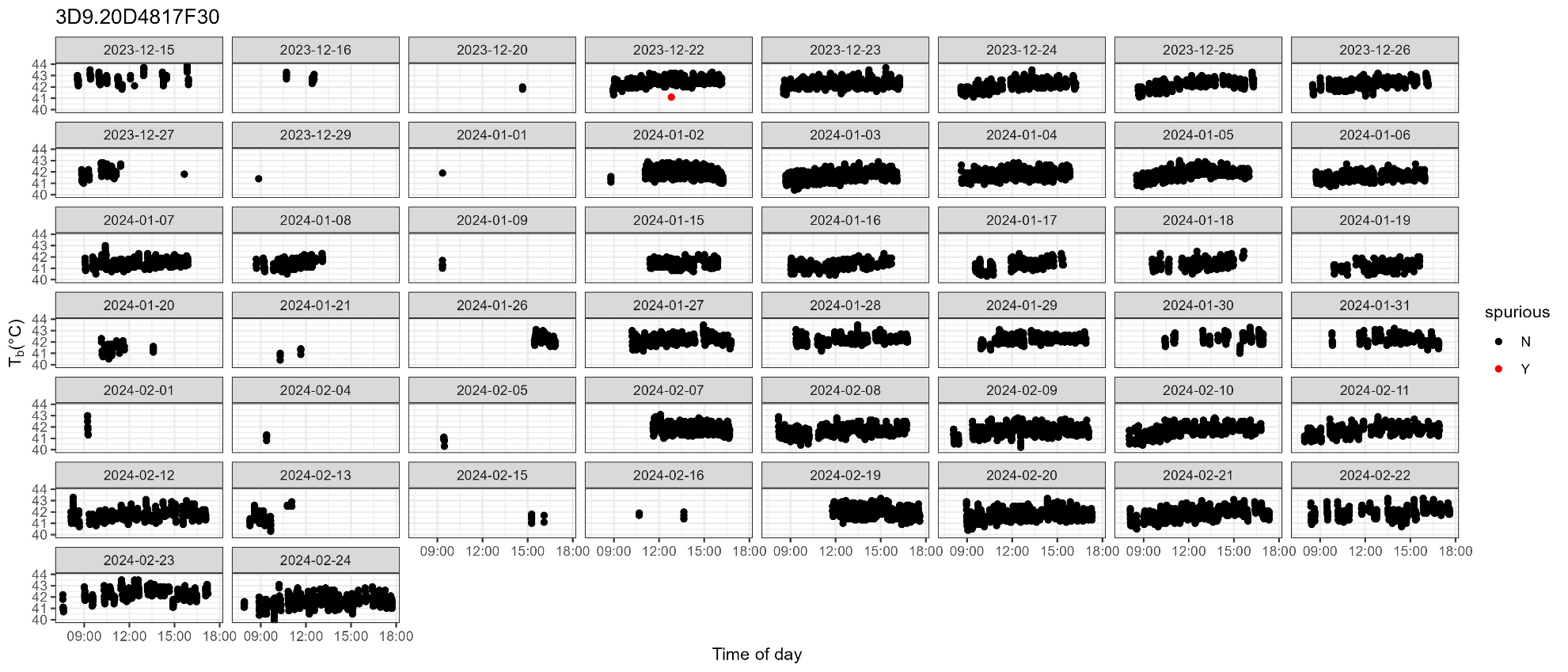


**Figure S22.** Daily plots of subcutaneous body temperatures (T _sub_) in relation to time of day for individual 3D9.20D4817F30 while they were detected at the thermal feeder during the food manipulation experiment. Black dots represent T_sub_ detections that were not identified as spurious while red dots represent T_sub_ detections that were identified as spurious.


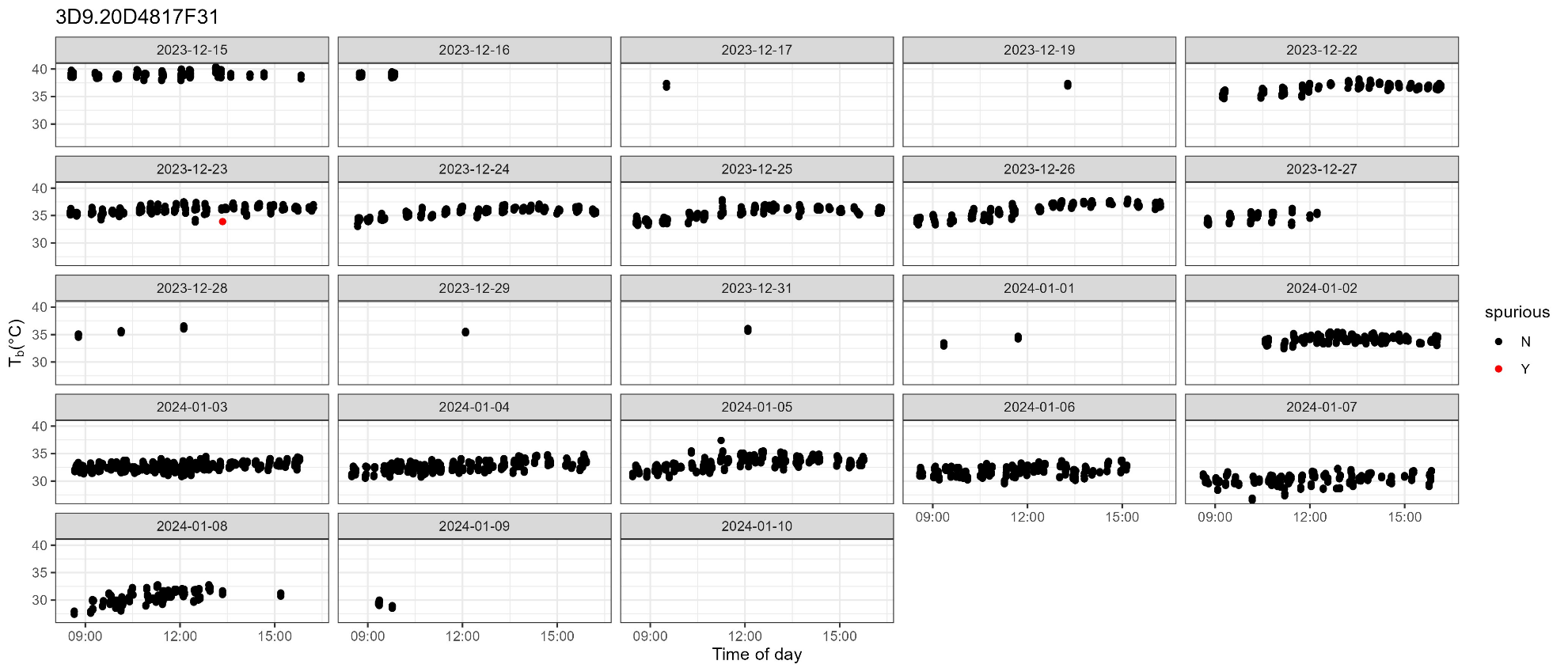


**Figure S23.** Daily plots of subcutaneous body temperatures (T _sub_) in relation to time of day for individual 3D9.20D4817F31 while they were detected at the thermal feeder during the food manipulation experiment. Black dots represent T_sub_ detections that were not identified as spurious while red dots represent T_sub_ detections that were identified as spurious.

# **Supplementary References**

Chaplin, S.B. 1974. Daily energetics of the black-capped chickadee, *Parus atricapillus*, in winter. J. Comp. Physiol., A*.* 89, 321-330.

Chaplin, S.B. 1976. The physiology of hypothermia in the black-capped chickadee, *Parus atricapillus*. J. Comp. Physiol., B*.* 112, 335-344.

Chaui-Berlinck, J.G., Alves Monteiro, L.H., Navas, C.A. & Bicudo, J.E.P.W. 2002. Temperature effects on energy metabolism: A dynamic system analysis. Proceedings of the Royal Society B: Biological Sciences*.* 269, 15-19.

Cooper, S.J. & Swanson, D.L. 1994. Seasonal acclimatization of thermoregulation in the black-capped chickadee. The Condor*.* 96, 638-646.

Hawkshaw, D.M., Wijmenga, J.J. & Mathot, K.J. 2025. Individual variation in diurnal body temperature and foraging activity in overwintering black-capped chickadees (*Poecile atricapillus*). J. Therm. Biol. 127, 104059.

Schmidt-Nielsen, K. 1997. *Animal physiology: Adaptation and environment.*  Cambridge University Press.

Scholander, P.F., Hock, R., Walters, V. & Irving, L. 1950. Adaptation to cold in arctic and tropical mammals and birds in relation to body temperature, insulation, and basal metabolic rate. The Biological Bulletin*.* 99, 259-271.

Stoffel, M.A., Nakagawa, S. & Schielzeth, H. 2017. Rptr: Repeatability estimation and variance decomposition by generalized linear mixed-effects models. Methods Ecol. Evol. 8, 1639-1644.
